# Fungal attack on archaeological wooden artefacts in the Arctic—implications in a changing climate

**DOI:** 10.1038/s41598-020-71518-5

**Published:** 2020-09-03

**Authors:** Nanna Bjerregaard Pedersen, Henning Matthiesen, Robert A. Blanchette, Gry Alfredsen, Benjamin W. Held, Andreas Westergaard-Nielsen, Jørgen Hollesen

**Affiliations:** 1 Institute of Conservation, Schools of Architecture, Design and Conservation, The Royal Danish Academy of Fine Arts, Esplanaden 34, 1263 Copenhagen K, Denmark; 2 Environmental Archaeology and Materials Science, National Museum of Denmark, I.C. Modewegsvej, Brede, 2800 Kgs. Lyngby, Denmark; 3 Department of Plant Pathology, University of Minnesota, 1991 Upper Buford Circle, 495 Borlaug Hall, St. Paul, MN 55108 USA; 4 Division of Forestry and Forest Resources, Norwegian Institute of Bioeconomy Research, Høgskoleveien 8, 1433 Ås, Norway; 5 Department of Geosciences and Natural Resource Management, Center for Permafrost (CENPERM), University of Copenhagen, Øster Voldgade 10, 1350 Copenhagen K, Denmark

**Keywords:** Microbiology, Climate sciences

## Abstract

Climate change is expected to accelerate the microbial degradation of the many extraordinary well-preserved organic archaeological deposits found in the Arctic. This could potentially lead to a major loss of wooden artefacts that are still buried within the region. Here, we carry out the first large-scale investigation of wood degradation within archaeological deposits in the Arctic. This is done based on wooden samples from 11 archaeological sites that are located along a climatic gradient in Western Greenland. Our results show that Ascomycota fungi are causing extensive soft rot decay at all sites regardless of climate and local environment, but the group is diverse and many of the species were only found once. *Cadophora* species known to cause soft rot in polar environments were the most abundant Ascomycota found and their occurrence in native wood samples underlines that they are present locally. Basidiomycota fungi were also present at all sites. In the majority of samples, however, these aggressive and potentially very damaging wood degraders have caused limited decay so far, probably due to unfavorable growth conditions. The presence of these wood degrading fungi suggests that archaeological wooden artefacts may become further endangered if climate change leads to more favorable growth conditions.

## Introduction

Archaeological deposits in Greenland often have a high abundance of wooden artefacts that primarily derive from Canadian or Siberian driftwood^1^. Even though only a fraction of the country’s archaeological deposits have been excavated, numerous spectacular finds have been made and the potential for further discoveries is anticipated to be high^2,3^. The buried wooden artefacts may, however, be under serious threat from climate change. Rising air temperatures cause longer periods of ground thawing and altered precipitation patterns^4,5^, and this may result in drying of near-surface layers and thereby increasing oxygen availability. Both factors are expected to accelerate the degradation of buried organic materials^6,7^ and lead to a significant reduction in the number of well-preserved archaeological deposits within the next century^7–9^.

Currently very little is known about the microbial and fungal communities in Arctic archaeological deposits and consequently it is difficult to foresee where and when archaeological wooden artefacts will be increasingly degraded following climatic changes. Studies of non-archaeological wood show that slow growing Ascomycota causing soft rot are the most common wood degraders in Polar Regions^10–15^. Soft rot fungi degrade the secondary cell wall but leave behind the compound middle lamella (CML)^16,17^. This results in preservation of a skeleton of the wood structure but with significant loss of strength. However, as long as the wood is kept waterlogged or frozen, the 3D structure and surface details of the wood may be well preserved^18^. Wood-inhabiting Basidiomycota fungi are also known to be present in the Polar Regions^19–22^. Basidiomycetes cause white and brown rot, which result in extensive damage after a relatively short time when the environmental conditions are suitable. This more aggressive degradation is fatal for archaeological wooden artefacts since all cell wall layers in the wood structure are degraded and consequently artefacts can be totally disintegrated^12,23–25^.

To date, the degradation of buried archaeological wooden artefacts has only been studied at one site in the Arctic^6^. Here optical microscopic analyses showed that archaeological wood samples taken from the permafrost were almost perfectly preserved, whereas clear signs of degradation by soft rot and a significant loss of mass were observed in samples taken from the active layer, which is the upper part of the soil that thaws every summer^6^.

Here we perform the first large-scale assessment of the distribution of wood degrading microbial communities associated with archaeological wooden artefacts in the Arctic. This is done based on samples of wooden artefacts collected from 11 contrasting archaeological deposits located along a north/south climatic gradient in Western Greenland. The aim is to determine the main types of wood degraders in the active layer and to identify potential connections between climatic conditions and distribution of the various wood decay fungi.

## Results

### Study sites and samples

**Figure 1:**
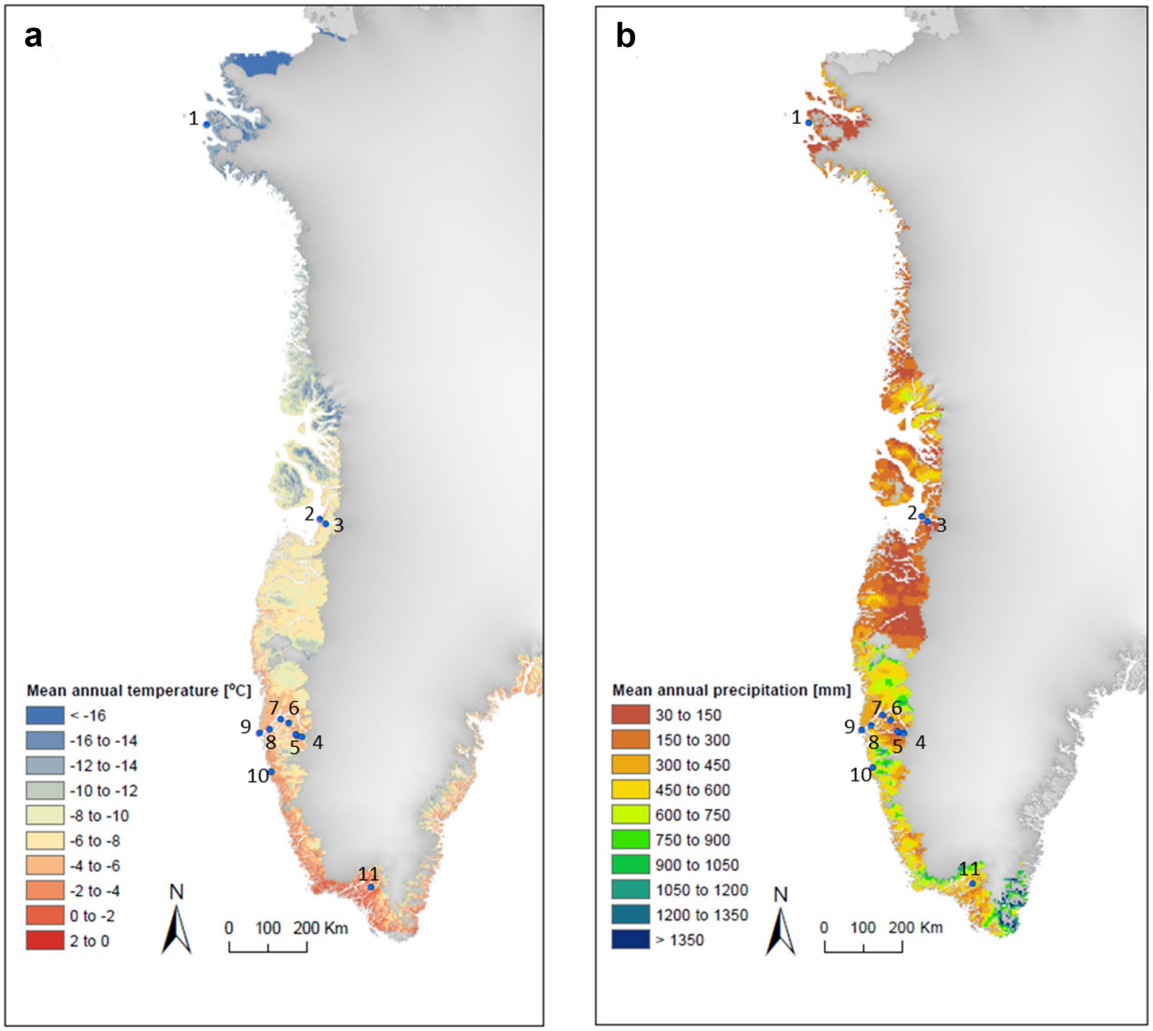
(**a**) Mean annual temperature derived from cloud-corrected MODIS MOD11A1 daily land surface temperatures, gap-filled with surface temperature from MAR v3.8. (see Hollesen et al.^7^ for site specific validation of the temperature data). (**b**) Mean annual precipitation consists of the annual mean of rain plus snow in mm water (equivalents). The data origins from daily values from the regional climate model MAR v3.8. The name and position of each site is given in Table 1. Figure was generated by Andreas Westergaard-Nielsen in ArcMap 10.3 (Environmental Systems Research Institute (ESRI) https://www.esri.com/software/arcgis), using the Layout View panel.

Our wooden samples from the 11 different archaeological sites (Fig. 1, Table 1) span over the cultures of Saqqaq (2,500–800 BC), Dorset (300BC–600AD), Thule (1,300AD–present), and the Norse settlers (985–1,450 AD). The sites were chosen in order to include samples from different cultures and time periods. The study sites are located along a north/south gradient with respect to air temperatures and soil temperatures (Fig. 1a) and are furthermore exposed to very different precipitation regimes (Fig. 1b). The variation in the mean annual thawing degree days (TDD) and the duration of the thaw period also varies greatly between the sites (Table 1).

**Table 1:** Geographical position of the 11 archaeological sites in addition to mean annual thawing degree days (TDD) and the duration of the thaw period at the soil surface.

Site no	Site	Latitude	Longitude	Cultural phases	Mean annual TDD (°C)^a^	Duration of thaw period (days)^b^
1	Nuulliit	76.80295	− 70.81849	Indepence 1, Late Dorset, Thule	537	95
2	Sermermiut	69.20172	− 51.12855	Saqqaq, Dorset, Thule	680	114
3	Qajaa	69.12777	− 50.70622	Saqqaq, Dorset, Thule	419	122
4	Austmannadal, (V53d)	64.21992	− 49.832758	Norse	1,236	133
5	Kilaarsarfik (Sandnes)	64.24264	− 50.182338	Saqqaq, Dorset, Norse	1,351	136
6	Iffiartarfik	64.46319	− 50.651597	Norse, Thule, Colonial (Hernnhut, Moravian)	1,215	151
7	Qoornoq	64.53314	− 51.089226	Norse, Thule, Colonial, Saqqaq (at nearby Tuapagssuit),	798	129
8	Ersaa	64.25176	− 51.602684	Thule, Colonial	910	144
9	Kangeq	64.10958	− 52.054651	Saqqaq, Thule, Colonial	648	144
10	Kangerdluarssugssuaic	63.30012	− 51.10244	Thule, Colonial	834	133
11	Gardar (Igaliku)	60.98646	− 45.42144	Norse	1,265	154

^a^Mean annual thawing degree days (TTD) from 2001 to 2016 based on cloud-corrected and gap-filled MODIS MOD11A1 land surface temperatures.

^b^Mean annual number of consecutive days with positive (> 0 °C) temperatures from 2001 to 2016. Based on the same input data as (1).

The site numbers refer to location on the map in Fig. 1.

Excavations and test trenching were carried out at nine study sites in the period 2009–2016. In total 45 archaeological wooden samples were collected from the active layers at these sites (referred to as buried archaeological samples) (Supplementary Table S1 online). When a large number of samples were available at a site, the most degraded wooden artefacts were sampled in order to study the worst case state of preservation. In addition, 21 archaeological wooden samples that were partly exposed to ambient air due to either erosion or prior archaeological excavations (in old profiles) were sampled at three sites (referred to as exposed archaeological samples) (Supplementary Table S1 online). Exposure to ambient air is expected to accelerate the microbial decay and possible favour wood-inhabiting Basidiomycota fungi. The samples therefore served as worst-case scenario for wood preservation associated with the active layer at the sites. Six samples of exposed historical wood were collected directly on the soil surface at two sites (referred to as exposed historical samples). The historical samples originate from imported conifer species and were left at the two sites approximately in 1970 and 1950. These samples served as measure/indicator of fungi associated with exposed non-native and non-driftwood conifer. Furthermore, 12 samples of native dead *Salix glauca* L. were collected directly on the soil surface at four sites (referred to as native dead samples). These samples served as an indicator for local fungi associated with dead native grown hardwood shrubs.

### Wood identification

We determined wood genera/species for each wood sample by examining the anatomical characteristics with transmission light microscope. Results show that 87% of the 45 buried archaeological wood samples were conifers of the genera *Picea* (51%), *Larix* (18%) and *Pinus* (4%) (Supplementary Table S1 online). Some of the samples (13%) were heavily decayed and could only be identified as conifer wood. Furthermore, the distinction between *Picea* and *Larix* was somewhat uncertain since these two wood genera have great anatomical resemblance and can only be distinguished with certainty by differences in bordered pitting in ray tracheids^26^. However, the often advanced stages of microbial decay made pit anatomy uncertain. The remaining 11% of the buried archaeological samples were *Salix*. The 21 exposed archaeological wood samples were all conifer of the genera *Picea* (38%), *Larix* (43%) and *Pinus* (19%), and the exposed historical wood samples were of the genera *Picea* (33%) and *Pinus* (67%) (Supplementary Table S1 online).

### Visual decay analysis

**Figure 2:**
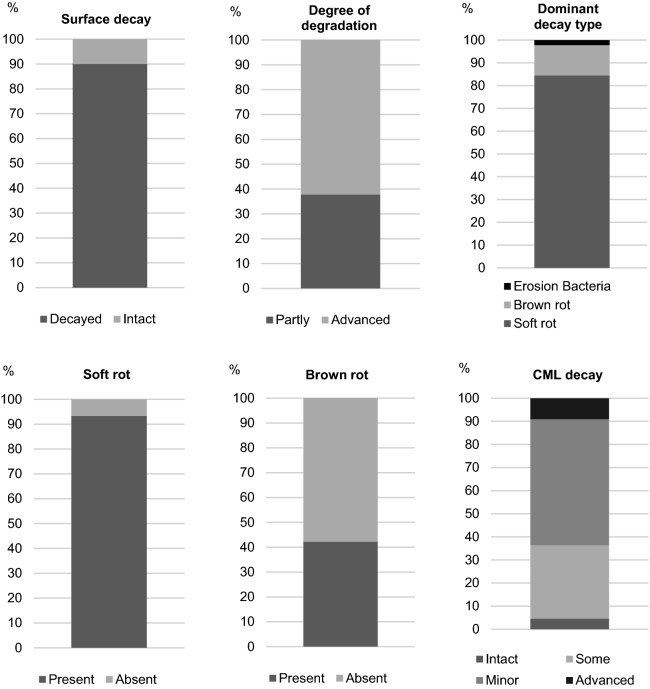
Results of visual decay analysis of the 45 buried archaeological wood samples unearthed from the active layer at nine archaeological sites shown as: Percentage of macroscopic determination of surface decay and microscopic determination of the dominating decay types, the presence and absence of soft rot and brown rot, the degree of degradation, and the degree of compound middle lamella (CML) decay.

**Figure 3:**
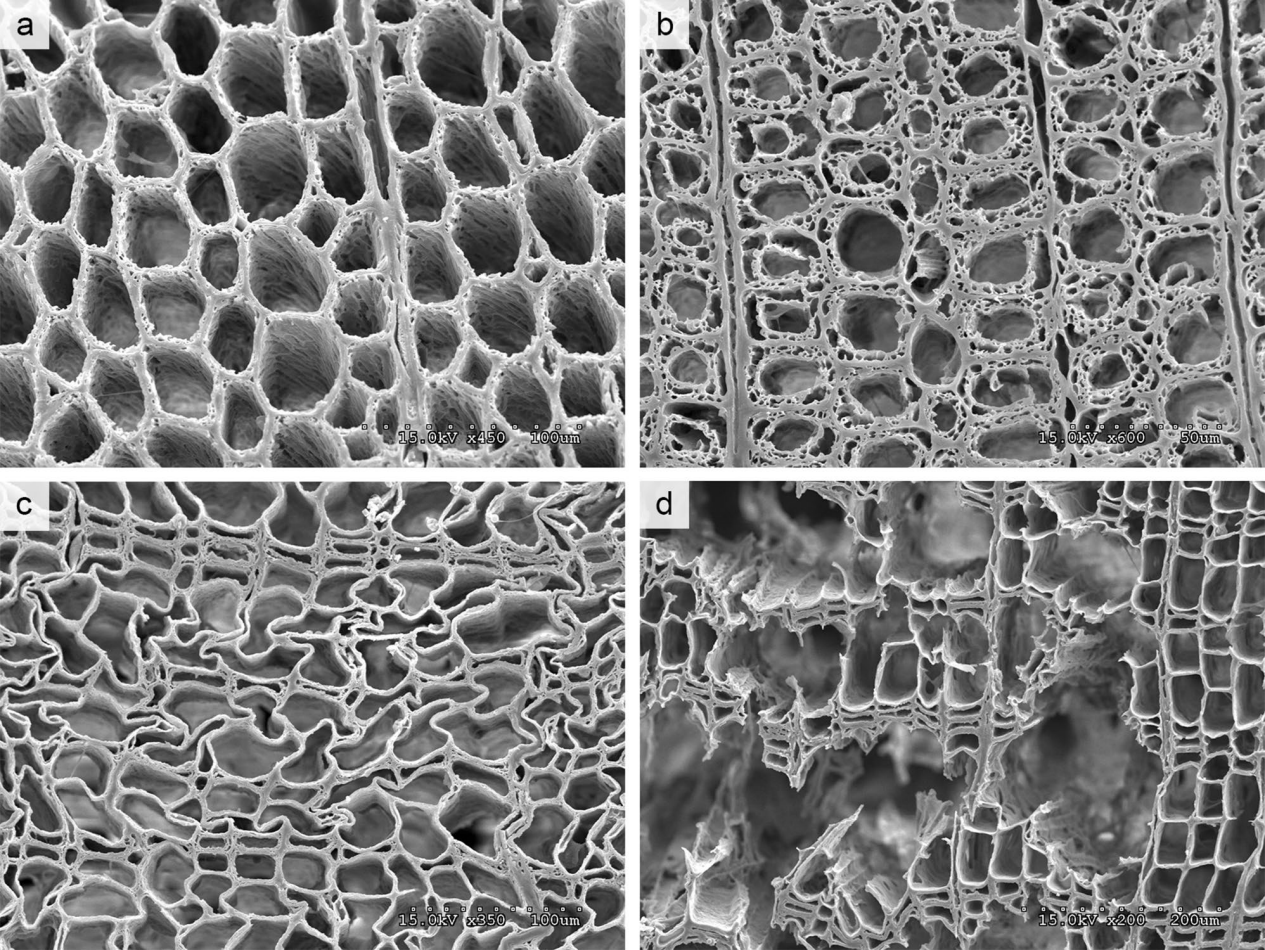
Scanning electron images of typical decay patterns in the collected sample material. Advanced soft rot decay (**a**, **b** sample 521 and 508 respectively), localized areas of brown rot decay (**c** sample 507), pockets where all cell wall layers were removed including the compound middle lamella (**d** sample 507). Benjamin W. Held generated the images.

The state of preservation of the buried archaeological wood samples was first evaluated by macroscopic examination of surface decay (Fig. 2). This revealed that 91% of the samples had some distortion of the surface which in itself entails lower scientific value as tool marks and surface decoration may be affected. Subsequently, the buried wood samples were evaluated by microscopic examination of cross sections cut from the surface towards the inner part of the samples to determine the types of decay present, the dominant decay type present, and the degree of degradation. All but four samples were decayed by soft rot which was the dominant decay type in 84% of the samples (Figs. 2, 3a,b). Brown rot was found in 42% of the buried archaeological samples, but was only the dominant decay type in 13% of the samples (Figs. 2, 3c). Only one of the buried archaeological samples was attacked by selective white rot. However, all but two samples had areas within the wood structure where all cell wall material, including the compound middle lamella, was missing leaving small holes in the material along with the otherwise dominating soft rot decay (Fig. 3d). This decay pattern could result from pockets of simultaneous white rot but the decay of the CML could also result from mechanical damage due to thaw/freeze cycles of the fragile material heavily decayed by soft rot. The degree of degradation was generally high with 62% of the samples in an advanced state of decay. Visual decay analysis of the 21 exposed archaeological wood samples and the six exposed historical wood samples showed decay patterns that were very similar to the ones found in the buried archaeological samples (Supplementary Fig. S2 and S3 online).

### DNA identification of fungi isolated from sampled wood material

**Figure 4:**
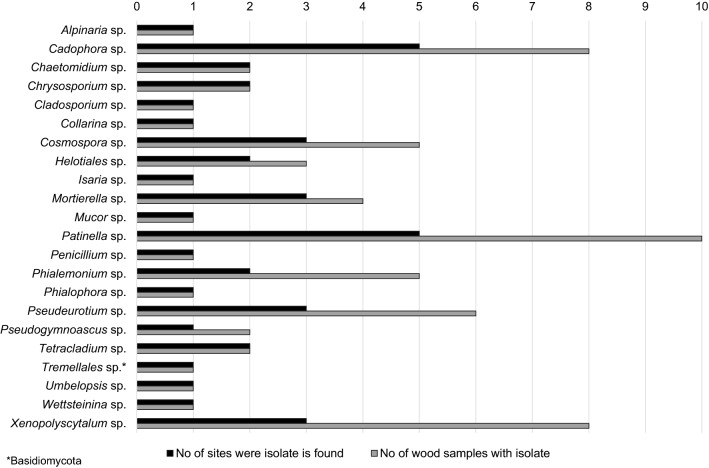
Identified filamentous fungal isolates grown from buried archaeological wood samples collected at nine archaeological sites. The figure specifies the number of unique wood samples from where the filamentous fungal isolates were grown and the number of sites where the isolates were found.

Incubation of wood samples on culture media followed by isolation of fungal mycelia and DNA sequencing for identification revealed a diverse group of Ascomycota as the dominant fungal type, with many of the species found only once (Supplementary Table S4 online). Among the Ascomycota found, the wood degrading soft rot fungus *Cadophora* sp. were more frequently isolated than other genera. *Cadophora* sp. were found at eight different sites, in 15 different wood samples, and in all four types of sample material (buried and exposed archaeological wood, exposed historical wood, and native dead wood). Other Ascomycota fungi known to cause soft rot in wood were found at seven different sites in a smaller number of wood samples (all sample types) including *Chaetomium*, *Cladosporium*, *Lecythophora*, *Leptodontidium*, and *Phialophora*. Strains known to cause soft rot in wood were not found at Nuulliit, Kilaarsarfik, and Kangerdluarssugssuaic, even though soft rot was observed in the wood samples. Ascomycota fungi found in a high number of samples (in eight to 11 wood samples at three to six different sites) includes *Penicillium*, *Patinella*, *Xenopolyscytalum, Pseudeurotium*, and *Phialemonium*. However, none of these five Ascomycota are known to cause soft rot in wood. *Xenopolyscytalum, Patinella*, and *Phialemonium* were only found in archaeological wood samples, and *Pseudeurotium* was found in the archaeological wood samples except for one isolated from a wood sample of exposed historical wood (Fig. 4 and Supplementary Fig. S5–S7 online).

Only a very limited number of filamentous Basidiomycota were isolated with most of the species found only once (Fig. 4, Supplementary Table S4 and Fig. S5–S7 online). One buried archaeological wood sample contained *Tremellales*, two exposed archaeological wood samples from two different sites contained *Hohenbuehelia* and *Lentinellus*, two exposed historical samples from two different sites contained *Dacrymyces* and an unknown Basidiomycota species, and four native dead *Salix* samples contained *Fomes*, *Lentinellus*, and *Mycena*.

**Figure 5:**
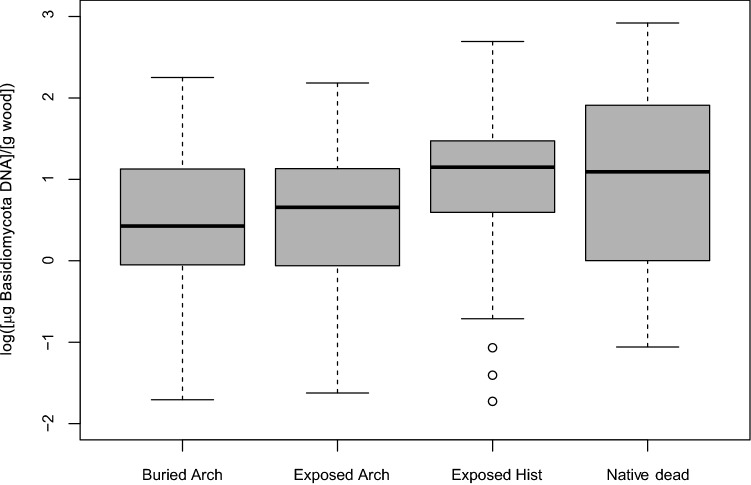
Median, upper and lower quartiles for quantitative Basidiomycota DNA determination in 64 wood samples with three biological replicas of each wood sample and three technical replicas of each sample replica. Data is shown at logarithmical scale of μg Basidiomycota DNA per gram wood. Whiskers show maximum and minimum values within 1.5 times the interquartile range and circles show values outside 1.5 times the interquartile range.

These findings only represent cultures that are alive or able to germinate under the given growth conditions. We therefore also performed extractions of gDNA followed by real-time PCR DNA analysis in order to obtain quantitative determination of Basidiomycota DNA in the sample material. gDNA extraction was performed directly from 29 of the buried archaeological wood samples, 15 of the exposed archaeological wood samples, all six exposed historical wood samples, and all 12 native dead *Salix* samples. The results show that all wood sample categories and wood from all sample sites contained Basidiomycota DNA but with a very variable concentration (Fig. 5). Only very few sub samples did not contain any Basidiomycota DNA (11 of the buried archaeological samples and one native dead *Salix* sample). The samples of native dead *Salix* and exposed historical wood contained notably more Basidiomycota DNA than both types of archaeological wood samples (exposed and buried) (Fig. 5). The quantification method is not selective towards wood decaying Basidiomycota and will therefore include quantification of Basidiomycota yeast and mycorrhiza that do not degrade wood, if present. Therefore, gDNA extracts with a high-quality melting curve were used for sequencing for fungal species identification. The 39 analysed subsamples (21 different wood samples from seven sites including all sample types) revealed that all the identified Basidiomycota fungal genera are from genera that are known to cause wood decay (Supplementary Table S8 and S9 online).

## Discussion

We investigated 66 archaeological samples of wood (buried or exposed) from 11 different archaeological sites in Western Greenland. The majority of samples (93%) were species of conifer that have not grown naturally in Greenland during at least the Holocene^27^. Not surprisingly, our results thereby underline the importance of driftwood as a source of raw material.

Ascomycota was the dominant decay type in more than 80% of the samples, not only in the buried archaeological wood, but also in the exposed archaeological samples and in the historical wood samples exposed directly on the ground for approximately 50 or 90 years. In most cases, the observed soft rot decay was very extensive. Two thirds of both the buried and the exposed samples were decayed to an advanced stage where the entire secondary cell wall was decayed. We specifically targeted the most degraded wooden artefacts at each site and thus our results do not reflect the average state of preservation but rather a worst-case scenario. Nevertheless, they clearly confirm that Ascomycota are readily abundant in Greenland’s archaeological deposits and that soft rot may cause significant damage to wooden artefacts in all parts of the country and across a substantial climatic gradient. *Cadophora* species were the most abundant of all the Ascomycota. It was found at eight of the 12 locations sampled and species of *Cadophora* are known to cause soft rot in many different polar environments^12,14,21,28,29^. Their high frequency of isolation in our study suggests that they are common and likely represent an important group of indigenous wood decay fungi well adapted to the extreme Arctic environment that attack archaeological and historic woods in Greenland. We also found a high frequency of *Patinella* (five sites), *Xenopolyscytalum* (four sites), *Pseudeurotium* (four sites), and *Phialemonium* (three sites) almost exclusively associated with the archaeological sample material buried or partly buried in the soil. These genera are rarely encountered^30,31^ but due to the high frequency, the close association to the buried material, and the advanced soft rot decay of the sample material from all sites, it is speculated that these species could also be causing soft rot decay in the archaeological wood material. Additional research is needed to determine their decay potential.

Driftwood is decayed by a rich variety of soft rot fungi^14^ and may already be colonized during waterborne travel or when lying at the coastline. This means that the wooden samples could have been colonized with soft rot when first picked up by humans centuries or even millennia ago. However, five of the six exposed historical (non-driftwood) samples and the exposed native dead *Salix* samples were also dominated by soft rot. The occurrence of *Cadophora* in native wood samples in this study underlines that they are present locally and do not need to be imported with the wood. Thereby our results indicate that soft rot fungi are present in surface layers of Arctic soils and readily colonize any wood whether it is historic or modern from native and non-native trees and shrubs. This is supported by studies by Blanchette et al.^14^ and Jurgens et al.^29^. Blanchette et al.^14^ show that the majority of fungi found in driftwood at sites in Greenland, Iceland, and the Siberian Lena Delta are similar to the native population. Jurgens et al.^29^ show that even non-mineralized fossil wood found in proglacial soil at Ellesmere Island, Canada is decayed by local soft rot fungi when in direct soil contact. We therefore conclude that the major source of these fungi are from indigenous populations, and only few fungi might have travelled large distances with the driftwood or were spread over large distances by humans or animals. The studied wood decaying fungi are part of the natural degradation process that has evolved in the Arctic over time. The fungi utilize carbon sources in the environment weather it is woody plants like Arctic willow or driftwood that arrives from great distances away. The wood is colonized from airborne spores or from direct contact with fungal mycelium in soils.

Our quantitative DNA analysis showed that Basidiomycota DNA was present in nearly all wood samples but in very variable amounts. Presence of wood decaying Basidiomycota was confirmed in our visual decay analysis. In total 42% of the buried archaeological samples showed signs of brown rot decay and 40% of the samples had some or advanced CML decay, which could result from simultaneous white rot decay. In addition, Basidiomycota associated with wood decay were found in a smaller portion of the samples selected for fungal species identification. This confirms that wood decaying Basidiomycota are abundant in Greenland’s archaeological deposits and these fungi pose a potential serious risk to wooden cultural heritage objects along the coastline of Western Greenland since they can cause extensive decay and destroy the wood.

It cannot be excluded that the observed Basidiomycota DNA originate from dead fungi and that the observed decay took place a long time ago when the wood was still exposed on the surface. With time and sustained deposition, the wood was buried and exposed to an environment with less oxygen and eventually the Basidiomycota fungi were outcompeted by the Ascomycota fungi. We found a notably higher average amount of Basidiomycota DNA in our replicates of natural grown dead *Salix* and exposed historical wood than in the archaeological wood from the active soil layer of the archaeological deposit (both buried and exposed). This suggests that the archaeological wood is more protected against Basidiomycota than wood exposed directly to the soil surface. Ascomycetes are known to tolerate higher moisture and lower oxygen levels than Basidiomycetes^24^. However, the exposed historical samples were only dominated by brown rot decay in one of the six samples; the five other samples were dominated by soft rot. Interestingly, the exposed archaeological samples did not have a higher average amount of Basidiomycota DNA than the buried archaeological samples, and the visual analysis showed very similar decay patterns between the two types of archaeological wood material. This indicates that despite exposure to ambient air and thereby less protection from the soil (lower oxygen level and more moisture), the wood is not in a markedly worse state of preservation than wood buried within the active layer.

Basidiomycota are aggressive fungi that may outcompete slower growing soft rot Ascomycota and therefore tend to become the dominant degrader when environmental conditions are conducive for their growth^32^. In this study, however, only very few wood samples were dominated by brown rot decay or by advanced CML decay that could originate from simultaneous white rot decay. Despite the fact that Basidiomycota are present at all the examined sites, Ascomycota seems to be better adapted to the conditions found within the archaeological deposits. Although the investigated samples were collected from sites that are exposed to different climatic conditions (Table 1), the results show no geographical trends in relation to both the severity of soft rot and the distribution of Basidiomycota wood degrading fungi. Therefore, it is not possible to pin-point the dominating driver for soft rot decay or the dominating limiting factor for Basidiomycota growth e.g. temperature, oxygen availability, nutrients, length of growth season, annual sum of thawing degree days or precipitation rates. This is probably because the fungal species interactions and fungal life cycles are a result of more complex dependence of several of these factors.

Air temperatures in Greenland are expected to increase with up to 5 °C within the next century^33^ leading to a marked increase in soil temperatures^7^ and a significant loss of permafrost^34^. We did not include archaeological wood samples from the permafrost layer in this investigation, but previous studies have shown that permafrost provides close to optimal preservation conditions for wooden artefacts^6^. Our results suggest that permafrost melt and exposure to the less stabile conditions within the active layer will likely lead to severe soft rot decay. It is not possible to estimate the rate at which the soft rot in our samples has occurred. However, previous incubation studies have shown that the degradation of wood is highly temperature dependent with rates increasing exponentially with increasing temperature^6^. The presence of early stages soft rot may not be critical, but advanced soft rot degradation results in a very fragile wood structure and little strength remaining in the wood. This means that the many wooden artefacts currently buried within the active layer may collapse and lose their scientific value if not kept moist. Thereby, wooden artefacts buried in areas where summer precipitation rates decrease, or the duration and frequency of drought periods increase may be especially vulnerable.

Furthermore, given the presence of Basidiomycota at all sites examined in this study, there is a potential risk that as environmental conditions change these fungi will become more active within the archaeological deposits causing serious losses in the future.

## Conclusion

In this study, we found that Ascomycota soft rot fungi were the dominant decay organisms at all 11 sites studied regardless of climate and local environment. Ascomycota had caused extensive soft rot decay in a high number of samples leaving a weak and vulnerable wood structure, however with preserved wood anatomical features. This first and foremost suggest that large parts of the wooden artefacts that are currently buried within the active layers of Greenland’s archaeological deposits may collapse and lose their scientific value if not kept moist. Secondly, it indicates that the uniquely well-preserved wooden artefacts that are currently buried within the permafrost will be attacked by Ascomycota and be subject to soft rot decay when the permafrost melts. However, the rate at which the soft rot decay occurs is still largely unknown. Basidiomycota was also present at all 11 sites but appeared to be outcompeted by Ascomycota in the great majority of the samples. Basidiomycota decay destroys the anatomical features of the wood and thereby the scientific value of the artefacts. Currently, multiple factors are most likely limiting the growth of the Basidiomycota wood degraders, but if growth conditions become more favourable with a changing environment, the archaeological wooden artefacts could be further endangered by these more aggressive wood destroying fungi.

## Methods

### Sampling of wood material

In total 45 buried archaeological samples, 21 exposed archaeological samples, six exposed historical samples, and 12 native dead *Salix glauca* samples were collected during field campaigns in the years 2009 and 2012–2016. Samples collected in 2014–2016 were placed in sterile bags and kept below freezing during transport and storage until investigated in the laboratory. Samples collected in 2009, 2012 and 2013 were collected in normal PE sample bags and kept frozen until investigated in the laboratory (Supplementary Table S1).

### Meteorological conditions

Mean annual precipitation was computed based on snow and rain-fall in mm water equivalents from the regional climate model MAR v3.8^35^, using daily 5 km data. The climate model handles redistribution of the winter snowpack through the CROCUS modelling scheme. Surface temperatures were extracted from the satellite based MOD11A1 MODIS product, offering daily data at 1 km spatial resolution. This product was corrected for cloud cover^36^ to minimize a cold winter bias, and subsequently gap-filled with MAR v3.8 surface temperatures again at 5 km spatial resolution. Only MAR-data from terrestrial areas were used; sea- and glacier-classified areas were omitted.

### Visual decay analysis and wood identification

66 archaeological (buried and exposed) and six historical wood samples from 11 archaeological sites were visually inspected to determine if the wood surface was intact or decayed. Thin sections, hand cut with razorblade in transverse, radial and tangential longitudinal direction stained with 0.1% (w/w) safranin O (Sigma-Aldrich, MO, USA), were examined in bright field transmission microscope (Leica DM5000B). Wood genera were determined using anatomical characteristics^37^. Decay types were determined by examination of anatomical characteristics for the major types of wood decaying microorganisms^24,25,32,38,39^. The degree of degradation was determined by examination of cross section from surface to inner xylem and an average degree of degradation of each sample was categorised either as intact, partly decayed, or in an advanced stage of decay (Supplementary Methods S10 online). Nine buried archaeological samples were selected for further scanning electron microscopy analysis (Supplementary Methods S10 online).

### Culturing and identification of fungi

Five sub-samples approximately 1 × 1 × 0.5 mm^3^ were cut as biological replicas from 53 archaeological samples (buried or exposed), the six exposed historical wood samples, and 12 native dead exposed *Salix glauca* samples using aseptic techniques. The sub-samples were incubated on at least two and up to five different culture media including at least one Basidiomycota specific culture media (Supplementary Methods S10 online). The sub-samples were incubated at room temperature (20–22 °C) as previous studies have shown that filamentous fungi from polar environments are primarily psychrotrophs or mesotrophs and can grow above 20°C^11,12,20^. Pure cultures were obtained by transferring isolations to individual plates of malt extract agar (15.0 g malt extract and 15.0 g agar in 1L deionized water). DNA was extracted from pure culture plates using a CTAB extraction protocol and the internal transcribed spacer region (ITS) of rDNA was amplified, sequenced and processed for GenBank searches following that of Blanchette et al.^14^.

### Quantitative Basidiomycota DNA determination

Material preparation: Three biological replicas of approximately 100 mg were taken from 46 archaeological samples (buried or exposed), six exposed historical wood samples, and 12 native dead exposed *Salix glauca* samples with aseptic and DNA-clean (10% bleach) techniques preferably in three different areas with visual surface decay. Each sub-sample was taken 1–3 mm below the surface (to avoid surface contamination) and cut as fine as possible with a scalpel. Two Basidiomycota monocultures, *Rhodonia placenta* ((Fr.) Niemelä, K.H. Larss. & Schigel (syn. *Postia placenta*) strain FPRL 280 and *Trametes versicolor* ((L.) Lloyd) stain CTB 863 were grown aseptic on cellophane to produce mycelia for a standard curve. One PCR-clean steal ball was added to each 2 mL Eppendorf tube (with Araldite glue in the lid) and the samples were ball milled 2 min (Retsch MM300, Retsch Gmbh, Haan, Germany) at speed 30 (1/s) after instant freezing of tubes and the mill sample holders in liquid nitrogen. 40 wood subsamples were too wet to mill directly and were freeze dried (Heto Drywinner, DW 1, 0-60E) prior to milling.

DNA extraction: Using 40.0 mg aliquots of powdered wood material genomic DNA was isolated from each subsample with the DNeasy Plant Mini kit (Qiagen, Hilden, Germany) according to the manufacturer's instructions. 1.00 ng pGEM of an internal reference DNA composed of pGEM plasmid (pGEM-3Z Vector, Promega, Madison, WI, USA) was added to each subsample upon DNA isolation to normalize for the natural variation in extractability of DNA in wood^40^. The extracted DNA was eluted in 50 µL AE buffer. The quantity and quality of the extracted DNA was determined by analysing 2 µL eluted DNA with a UV–VIS micro volume spectrophotometer (NanoDrop 2000, Thermo Scientific, Waltham, MA, USA). Details about experimental sample dilution and preparation of standard curves are provided in Supplementary Methods S10 online.

Primer selection: Primer selection was based on Børja et al.^41^ and Pilgard et al.^42^ for Basidiomycota and the internal pGEM reference DNA were selected as previously reported by Coyne et al.^40^.

qPCR conditions: The quantity of Basidiomycota DNA (DNA_bas_) in each subsample was determined by quantitative polymerase chain reaction (qPCR). Amplifications of DNA_bas_ were performed in Fast SYBRGreen Master Mix (Applied Biosystems, Waltham, MA, USA) whereas pGEM was amplified in TaqMan Universal PCR Master Mix (Applied Biosystems, Waltham, MA, USA). The qPCR was performed with AB Applied Biosystems ViiA 7 (Applied Biosystems, Waltham, MA, USA) running with an initial activation step at 95 °C for 2 min followed by 45 PCR cycles running at 95 °C for 15 s followed by 1 min at 60 °C. A melting curve was determined at the end of cycling DNA_bas_ to get information on the quality of the PCR products in each sample. Ct values were determined with the Applied Biosystems ViiA 7, v1.2.1 software supplied with the instrument. The extent of amplification was calculated as a mean Cq value of two technical replicates for each sample and calculated into gDNA concentration via gDNA and pGEM standard curves.

### Identification of Basidiomycota in gDNA extracted directly from wood samples

The melting curves from the quantitative Basidiomycota gDNA determination revealed that 39 subsamples from 21 different wood samples had a high-quality melting curve with a single PCR product. The DNA extractions from these samples were used for sequencing for fungal species identification. Amplification was performed with the same 5.8sr forward primer and ITS4-X reverse primer as used for quantitative Basidiomycota DNA determination. Details about the mastermix is provided in Supplementary Methods S10 online. The DNA extractions from the selected subsamples were diluted 100 × except for three samples that were diluted 1,000 × . Each subsample was prepared by mixing 2.00 µL diluted isolated gDNA with the mastermix prior to amplification. In addition one blank sample was prepared by mixing 2.00 µL nuclease free water with the mastermix. Details about PCR amplification is provided in Supplementary Methods S10 online. 32 of the 39 run PCR products showed a band at the agarose gel and were selected for Sanger sequencing run at GATC Biotech (Konstanz, Germany). 20 of the sequenced PCR products were De Novo assembled using Geneious 7.0 (Biomatters, Auckland, New Zealand) to a consensus sequence from the forward and reverse sequence and the best nucleotide BLAST (U.S. National Library of Medicine) matched to GenBank sequences using, if possible, accessions from taxonomic publications was performed. PCR products from five subsamples could not be De Novo assembled but nucleotide BLAST on both the forward and reverse sequence gave match of the same genus. PCR products from five other subsamples gave a high quality forward or reverse sequence that could be BLAST but non or very low quality of the opposite sequence. PCR products from two subsamples did not have a high enough sequence quality for either De Novo assembling or nucleotide BLAST on either the forward or reverse sequence.

## Supplementary information

Supplementary Information 1.

## Data Availability

The datasets generated during and/or analysed during the current study are available from the corresponding author on reasonable request.

## References

[1] Hellmann L (2013). Tracing the origin of Arctic driftwood. J. Geophys. Res. Biogeosci..

[2] Hollesen J, Dawson T, Nimura C, López-Romero E, Daire M-Y (2017). Climate change and the preservation of archaeological sites in Greenland. Public Archaeology and Climate Change.

[3] Grønnow B (2017). The Frozen Saqqaq Sites of Disko Bay, West Greenland, Qeqertasussuk and Qajaa (2400–900 BC), Studies of Saqqaq Material Culture in an Eastern Arctic Perspective.

[4] ICPP*. Summary for Policymakers. Climate Change 2013. The Physical Science Basis. Contribution of Working Group I to the Fifth Assessment Report of the Intergovernmental Panel on Climate Change* (Cambridge, UK and New York, NY, USA, 2013).

[5] Kaufman DS (2009). Recent warming reverses long-term arctic cooling. Science.

[6] Matthiesen H, Jensen JB, Gregory D, Hollesen J, Elberling B (2014). Degradation of archaeological wood under freezing and thawing conditions—effects of permafrost and climate change. Archaeometry.

[7] Hollesen J (2019). Predicting the loss of organic archaeological deposits at a regional scale in Greenland. Sci. Rep..

[8] Elberling B (2011). Paleo-Eskimo kitchen midden preservation in permafrost under future climate conditions at Qajaa, West Greenland. J. Archaeol. Sci..

[9] Hollesen J (2012). The future preservation of a permanently frozen kitchen midden in western Greenland. Conserv. Manag. Archaeol. Sites.

[10] Held BW, Jurgens JA, Duncan SM, Farrell RL, Blanchette RA (2005). Assessment of fungal diversity and deterioration in a wooden structure at New Harbor, Antarctica. Polar Biol..

[11] Arenz BE, Blanchette RA (2009). Investigations of fungal diversity in wooden structures and soils at historic sites on the Antarctic Peninsula. Can. J. Microbiol..

[12] Blanchette RA (2010). An Antarctic hot spot for fungi at Shackleton's historic hut on Cape Royds. Microb. Ecol..

[13] Arenz BE, Held BW, Jurgens JA, Blanchette RA (2011). Fungal colonization of exotic substrates in Antarctica. Fungal Divers..

[14] Blanchette RA, Held BW, Hellmann L, Millman L, Büntgen U (2016). Arctic driftwood reveals unexpectedly rich fungal diversity. Fungal Ecol..

[15] Arenz BE, Blanchette RA (2011). Distribution and abundance of soil fungi in Antarctica at sites on the Peninsula, Ross Sea Region and McMurdo Dry Valleys. Soil Biol. Biochem..

[16] Daniel G, Nilsson T, Bruce A, Palfreyman JW (1998). Developments in the study of soft rot and bacterial decay. Forest Products Biotechnology.

[17] Kim YS, Singh AP (2000). Micromorphological characteristics of wood biodegradation in wet environments: a review. Iawa J..

[18] Pedersen, N. B., Björdal, C. G., Jensen, P. & Felby, C. *Stability of Complex Carbohydrate Structures. Biofuel, Foods, Vaccines and Shipwrecks*, Vol. 341 Special Publication (ed. Harding, S. E.) 160–187 (The Royal Society of Chemistry, London, 2013).

[19] Knudsen, H., Hallenberg, N. & Mukhin, V. A. A comparison of wood-inhabiting basidiomycetes from three valleys in Greenland. In *Arctic and Alpine Mycology 3–4. Proceedings of the Third and Fourth International Symposium on Arcto-Alpine Mycology* (eds. Orlando, P. & Laursen, G. A.) 133–145 (J. Cramer, Svalbard, 1993).

[20] Ludley KE, Robinson CH (2008). ‘Decomposer’ Basidiomycota in Arctic and Antarctic ecosystems. Soil Biol. Biochem..

[21] Held BW, Blanchette RA (2017). Deception Island, Antarctica, harbors a diverse assemblage of wood decay fungi. Fungal Biol..

[22] Mattsson J, Flyen A-C, Nunez M (2010). Wood-decaying fungi in protected buildings and structures on Svalbard. Agarica.

[23] Björdal CG, Nilsson T (2002). Waterlogged archaeological wood—a substrate for white rot fungi during drainage of wetlands. Int. Biodeterior. Biodegrad..

[24] Blanchette RA, Nilsson T, Daniel G, Abad A, Rowell RM, Barbour RJ (1990). Biological degradation of wood. Archaeological Wood Advances in Chemistry.

[25] Schwarze FWMR (2007). Wood decay under the microscope. Fungal Biol. Rev..

[26] Anagnost SE, Meyer RW, Dezeeuw C (1994). Confirmation and significance of Bartholin method for the identification of the wood of *Picea* and *Larix*. Iawa J..

[27] Normand S (2013). A greener Greenland? Climatic potential and long-term constraints on future expansions of trees and shrubs. Philos. Trans. R. Soc. B.

[28] Blanchette RA (2004). Wood-destroying soft rot fungi in the historic expedition huts of Antarctica. J. Appl. Environ. Microbiol..

[29] Jurgens JA, Blanchette RA, Filley TR (2009). Fungal diversity and deterioration in mummified woods from the ad Astra Ice Cap region in the Canadian High Arctic. Polar Biol..

[30] Baral H-O, Carter A (2013). *Patinella hyalophaea* Sacc.—rediscovered in New Brunswick, Canada. Ascomycete.org.

[31] Crous PW (2010). Persoonial reflections. Xenopolyscytalum Cros. gen. nov. *Fungal Planet 55*. Persoonia.

[32] Eriksson K-EL, Blanchette RA, Ander P (1990). Microbial and Enzymatic Degradation of Wood and Wood Components.

[33] 33IPCC. *Climate Change 2013: The Physical Science Basis. Contribution of Working Group I to the Fifth Assessment Report of the Intergovernmental Panel on Climate Change*. 1535 pp. (Cambridge University Press, Cambridge, 2013).

[34] Schuur EAG (2015). Climate change and the permafrost carbon feedback. Nature.

[35] Fettweis X (2017). Reconstructions of the 1900–2015 Greenland ice sheet surface mass balance using the regional climate MAR model. Cryosphere.

[36] Westergaard-Nielsen A, Karami M, Hansen BU, Westermann S, Elberling B (2018). Contrasting temperature trends across the ice-free part of Greenland. Sci. Rep..

[37] Schweingruber FH (1990). Microscopic Wood Anatomy/Mikroskopische Holzanatomie/Anatomie Microscopique du Bois: Structural Variability of Stems and Twigs in Recent and Subfossil Woods from Central Europe.

[38] Björdal CG, Daniel G, Nilsson T (2000). Depth of burial, an important factor in controlling bacterial decay of waterlogged archaeological poles. Int. Biodeterior. Biodegrad..

[39] Reinprecht L (2016). Wood Deterioration, Protection and Maintenance.

[40] Coyne KJ (2005). Improved quantitative real-time PCR assays for enumeration of harmful algal species in field samples using an exogenous DNA reference standard. Limnol. Oceanogr. Methods.

[41] Børja I, Alfredsen G, Filbakk T, Fossdal CG (2015). DNA quantification of basidiomycetous fungi during storage of logging residues. PeerJ.

[42] Pilgard A, Alfredsen G, Bjordal CG, Fossdal CG, Borja I (2011). qPCR as a tool to study basidiomycete colonization in wooden field stakes. Holzforschung.

