# Supplementary material for: Fungal attack on archaeological wooden artefacts in the Arctic—implications in a changing climate
Source: Sci Rep. 2020 Sep 3;10:14577. doi: 10.1038/s41598-020-71518-5 (PMC7471681; doi:10.1038/s41598-020-71518-5)
Supplement: Supplementary file 1 — Supplementary Information 1. [file 41598_2020_71518_MOESM1_ESM.pdf]

Supplementary material for the manuscript

Fungal attack on archaeological wooden artefacts in the Arctic - implications in a changing climate

Nanna Bjerregaard Pedersen[1,2*], Henning Matthiesen[2], Robert A. Blanchette[3], Gry Alfredsen[4], Benjamin W. Held[3], Andreas Westergaard-Nielsen[5], Jørgen Hollesen[2]

[1]Institute of Conservation, The Royal Danish Academy of Fine Arts, Schools of Architecture, Design and Conservation, Esplanaden 34, DK-1263 Copenhagen K, Denmark

[2]Environmental Archaeology and Materials Science, National Museum of Denmark, I.C. Modewegsvej, Brede, DK-2800 Kgs. Lyngby, Denmark

[3]Department of Plant Pathology, University of Minnesota, 1991 Upper Buford Circle, 495 Borlaug Hall, St. Paul, Minnesota, 55108 USA

[4]Division of Forestry and Forest Resources, Norwegian Institute of Bioeconomy Research, Høgskoleveien 8, N-1433 Ås, Norway

[5]Center for Permafrost (CENPERM), Department of Geosciences and Natural Resource Management, University of Copenhagen, Øster Voldgade 10, DK-1350 Copenhagen K, Denmark

*Corresponding author

E-mail: nbje@kadk.dk

Supplementary Table S1

Supplementary Table S1: Overview of collected wood samples including: Archaeological site, wood category, find context, wood genera/species, sampling year and sampling strategy

Site no.	Site	Wood category	Find context	Wood genera	Sampling year	Sample strategy
1	Nuulliit	Archaeological	Buried in active layer	*Larix*	2015	Aseptic
1	Nuulliit	Archaeological	Buried in active layer	*Picea abies*	2015	Aseptic
1	Nuulliit	Archaeological	Buried in active layer	*Picea abies*	2015	Aseptic
1	Nuulliit	Archaeological	Buried in active layer	*Picea abies*	2015	Aseptic
1	Nuulliit	Archaeological	Buried in active layer	*Picea abies*	2015	Aseptic
1	Nuulliit	Archaeological	Buried in active layer	*Picea abies*	2015	Aseptic
1	Nuulliit	Archaeological	Buried in active layer	*Picea abies*	2015	Aseptic
1	Nuulliit	Archaeological	Buried in active layer	*Salix*	2015	Aseptic
2	Sermermiut	Archaeological	Exposed in profile in active layer	*Larix*	2014	Aseptic
2	Sermermiut	Archaeological	Exposed in profile in active layer	*Picea abies*	2014	Aseptic
2	Sermermiut	Archaeological	Exposed in profile in active layer	*Picea abies*	2014	Aseptic
2	Sermermiut	Archaeological	Exposed in profile in active layer	*Larix*	2014	Aseptic
3	Qajaa	Archaeological	Buried in active layer	*Picea abies*	2009	-
3	Qajaa	Archaeological	Buried in active layer	*Pices abies*	2009	-
3	Qajaa	Archaeological	Exposed in profile in active layer	*Larix*	2014	Aseptic
3	Qajaa	Archaeological	Exposed in profile in active layer	*Larix*	2014	Aseptic
3	Qajaa	Archaeological	Exposed in profile in active layer	*Larix*	2014	Aseptic
3	Qajaa	Archaeological	Exposed in profile in active layer	*Larix*	2014	Aseptic
3	Qajaa	Archaeological	Exposed in profile in active layer	*Picea abies*	2014	Aseptic
3	Qajaa	Archaeological	Exposed in profile in active layer	*Pinus sylvestris*	2014	Aseptic
4	Austmannadal	Historical	Exposed on soil surface approximately 90 years	*Pinus sylvestris*	2012	-
4	Austmannadal	Historical	Exposed on soil surface approximately 90 years	*Picea abies*	2016	Aseptic
4	Austmannadal	Historical	Exposed on soil surface approximately 90 years	*Pinus sylvestris*	2016	Aseptic
4	Austmannadal	Native dead	Exposed on soil surface	*Salix glauca*	2016	Aseptic
4	Austmannadal	Native dead	Exposed on soil surface	*Salix glauca*	2016	Aseptic
4	Austmannadal	Native dead	Exposed on soil surface	*Salix glauca*	2016	Aseptic
5	Kilaarsafik (Sandnes)	Archaeological	Buried in active layer	Conifer	2012	-

Site no.	Site	Wood category	Find context	Wood genera	Sampling year	Sample strategy
5	Kilaarsafik (Sandnes)	Archaeological	Buried in active layer	*Salix*	2012	-
5	Kilaarsafik (Sandnes)	Archaeological	Buried in active layer	*Salix*	2012	-
5	Kilaarsafik (Sandnes)	Archaeological	Buried in active layer	Conifer	2016	Aseptic
5	Kilaarsafik (Sandnes)	Archaeological	Buried in active layer	Conifer	2016	Aseptic
5	Kilaarsafik (Sandnes)	Archaeological	Buried in active layer	*Picea abies*	2016	Aseptic
7	Qoornoq	Archaeological	Buried in active layer	*Larix*	2016	Aseptic
7	Qoornoq	Archaeological	Buried in active layer	*Picea*	2016	Aseptic
7	Qoornoq	Archaeological	Buried in active layer	*Pinus*	2016	Aseptic
7	Qoornoq	Native dead	Exposed on soil surface	*Salix glauca*	2016	Aseptic
7	Qoornoq	Native dead	Exposed on soil surface	*Salix glauca*	2016	Aseptic
7	Qoornoq	Native dead	Exposed on soil surface	*Salix glauca*	2016	Aseptic
2	Sermermiut	Archaeological	Exposed in profile in active layer	*Larix*	2014	Aseptic
2	Sermermiut	Archaeological	Exposed in profile in active layer	*Picea abies*	2014	Aseptic
2	Sermermiut	Archaeological	Exposed in profile in active layer	*Picea abies*	2014	Aseptic
2	Sermermiut	Archaeological	Exposed in profile in active layer	*Larix*	2016	Aseptic
2	Sermermiut	Archaeological	Exposed in profile in active layer	*Larix*	2016	Aseptic
2	Sermermiut	Archaeological	Exposed in profile in active layer	*Picea abies*	2016	Aseptic
6	Iffiartarfik	Archaeological	Buried in active layer	Conifer	2016	Aseptic
6	Iffiartarfik	Archaeological	Buried in active layer	NA	2016	Aseptic
6	Iffiartarfik	Archaeological	Buried in active layer	*Picea*	2016	Aseptic
6	Iffiartarfik	Native dead	Exposed on soil surface	*Salix glauca*	2016	Aseptic
6	Iffiartarfik	Native dead	Exposed on soil surface	*Salix glauca*	2016	Aseptic
6	Iffiartarfik	Native dead	Exposed on soil surface	*Salix glauca*	2016	Aseptic
8	Ersaâ	Archaeological	Buried in active layer	*Picea abies*	2016	Aseptic
8	Ersaâ	Native dead	Exposed on soil surface	*Salix glauca*	2016	Aseptic
8	Ersaâ	Native dead	Exposed on soil surface	*Salix glauca*	2016	Aseptic
8	Ersaâ	Native dead	Exposed on soil surface	*Salix glauca*	2016	Aseptic
9	Kangeq	Archaeological	Exposed in profile in active layer	*Picea abies*	2012	-
9	Kangeq	Archaeological	Exposed in profile in active layer	*Picea abies*	2012	-
9	Kangeq	Archaeological	Exposed in profile in active layer	*Picea abies*	2012	-
9	Kangeq	Archaeological	Buried in active layer	*Larix*	2012	-
9	Kangeq	Archaeological	Buried in active layer	*Picea abies*	2012	-
9	Kangeq	Archaeological	Buried in active layer	*Picea abies*	2012	-
9	Kangeq	Archaeological	Buried in active layer	*Picea abies*	2012	-

Site no.	Site	Wood category	Find context	Wood genera	Sampling year	Sample strategy
9	Kangeq	Archaeological	Exposed in profile in active layer	*Larix*	2014	Aseptic
9	Kangeq	Archaeological	Exposed in profile in active layer	*Picea abies*	2014	Aseptic
9	Kangeq	Archaeological	Exposed in profile in active layer	*Picea abies*	2014	Aseptic
9	Kangeq	Archaeological	Exposed in profile in active layer	*Picea abies*	2016	Aseptic
9	Kangeq	Archaeological	Exposed in profile in active layer	*Picea abies*	2016	Aseptic
9	Kangeq	Archaeological	Exposed in profile in active layer	*Picea abies*	2016	Aseptic
9	Kangeq	Archaeological	Exposed in profile in active layer	*Pinus sylvestris*	2016	Aseptic
9	Kangeq	Archaeological	Exposed in profile in active layer	*Pinus sylvestris*	2016	Aseptic
9	Kangeq	Archaeological	Exposed in profile in active layer	*Pinus sylvestris*	2016	Aseptic
9	Kangeq	Archaeological	Buried in active layer	*Larix*	2016	Aseptic
9	Kangeq	Archaeological	Buried in active layer	*Picea abies*	2016	Aseptic
9	Kangeq	Archaeological	Buried in active layer	*Picea abies*	2016	Aseptic
9	Kangeq	Archaeological	Buried in active layer	*Picea abies*	2016	Aseptic
9	Kangeq	Historical	Exposed on soil surface approximately 50 years	*Picea abies*	2016	Aseptic
9	Kangeq	Historical	Exposed on soil surface approximately 50 years	*Pinus sylvestris*	2016	Aseptic
9	Kangeq	Historical	Exposed on soil surface approximately 50 years	*Pinus sylvestris*	2016	Aseptic
10	Kangerdluarssugssuaic	Archaeological	Buried in active layer	Conifer	2012	-
10	Kangerdluarssugssuaic	Archaeological	Buried in active layer	Conifer	2012	-
10	Kangerdluarssugssuaic	Archaeological	Buried in active layer	*Larix*	2012	-
10	Kangerdluarssugssuaic	Archaeological	Buried in active layer	*Salix*	2012	-
10	Kangerdluarssugssuaic	Archaeological	Buried in active layer	*Salix*	2012	-
11	Gardar (Igaliku)	Archaeological	Buried in active layer	*Larix*	2013	-
11	Gardar (Igaliku)	Archaeological	Buried in active layer	*Larix*	2013	-
11	Gardar (Igaliku)	Archaeological	Buried in active layer	*Larix*	2013	-
11	Gardar (Igaliku)	Archaeological	Buried in active layer	*Picea abies*	2013	-
11	Gardar (Igaliku)	Archaeological	Buried in active layer	*Picea abies*	2013	-
11	Gardar (Igaliku)	Archaeological	Buried in active layer	*Pinus*	2013	-

Supplementary Figure S2

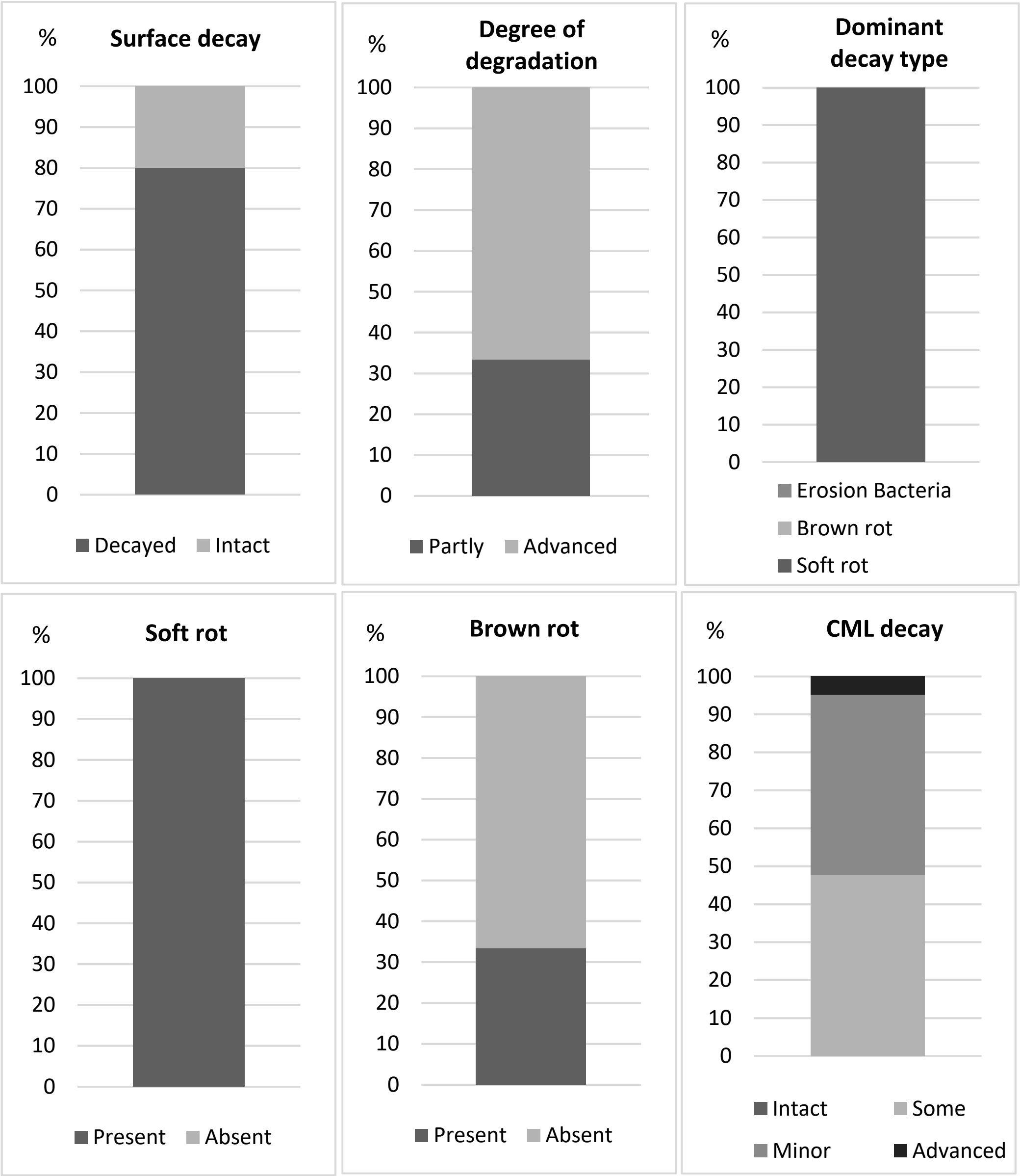

Supplementary Figure S2: Results of visual decay analysis of the 21 archaeological wood samples exposed to ambient air at three archaeological sites, shown as: Percentage of macroscopic determination of surface decay and microscopic determination of degree of degradation, the dominating decay types, the presence and absence of soft rot and brown rot, and the level of CML decay.

Supplementary Figure S3

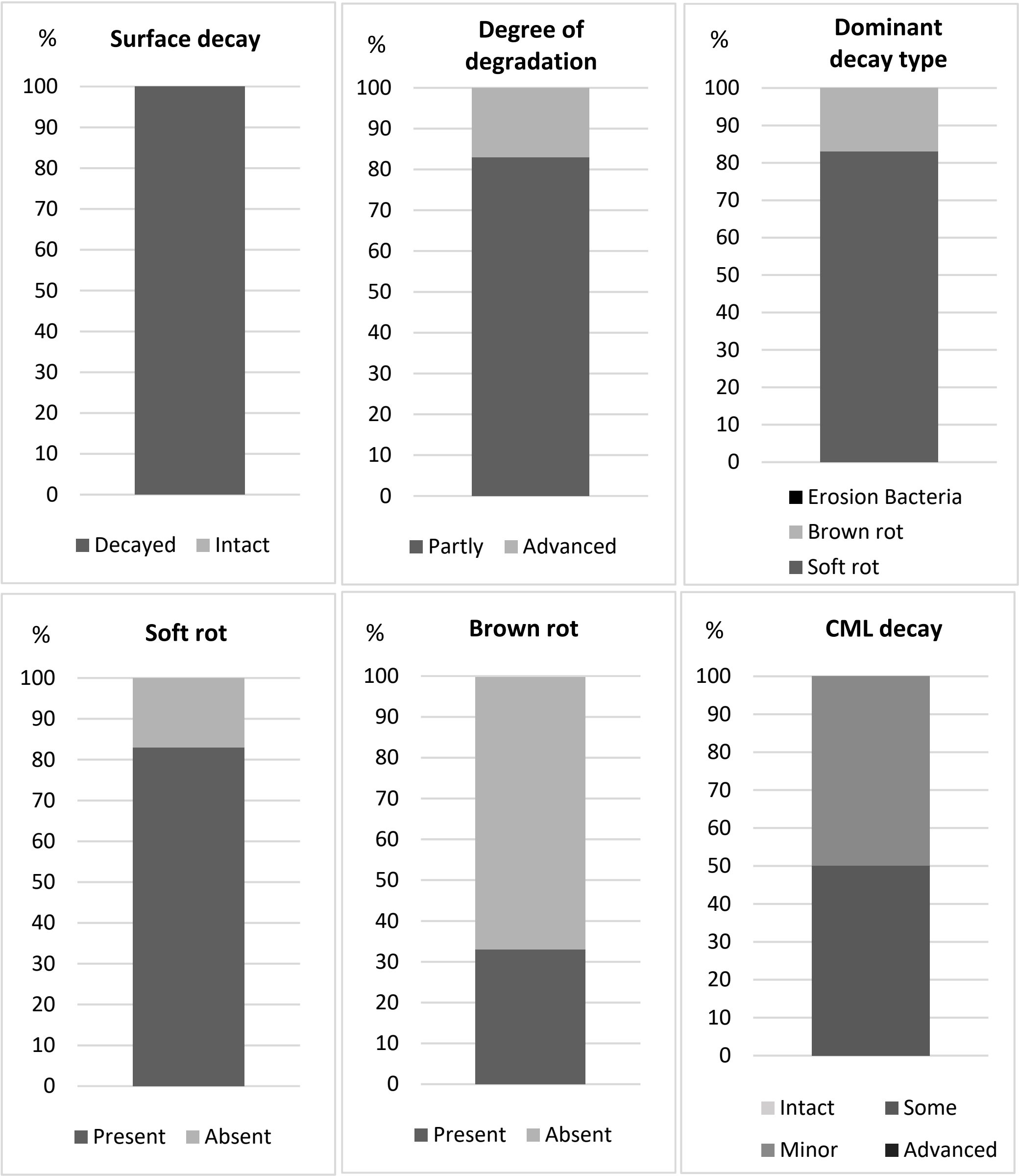

Supplementary Figure S3: Results of visual decay analysis of the six historical wood samples exposed to ambient air at two archaeological sites, shown as: Percentage of macroscopic determination of surface decay and microscopic determination of degree of degradation, the dominating decay types, the presence and absence of soft rot and brown rot, and the level of CML decay.

Supplementary Table S4

Supplementary Table S4: Fungi isolated from wood samples collected on archaeological sites in Western Greenland

Organism	Fungal type	Site	Isolate	Sample type	GenBank #
Alpinaria sp.	Ascomycota	Nuulliit	520-A	Buried Arch	MK163738
Basidiomycota sp.	Basidiomycota	Kangeq	623-D	Exposed Hist	MK163884
Basidiomycota sp.	Basidiomycota	Kangeq	623-E	Exposed Hist	MK163885
Cadophora sp.	Ascomycota	Austmannadal	570-A	Native dead *Salix*	MK163790
Cadophora sp.	Ascomycota	Ersaa	601-A	Buried Arch	MK163867
Cadophora sp.	Ascomycota	Ersaa	604-E	Native dead *Salix*	MK163797
Cadophora sp.	Ascomycota	Ersaa	604-F	Native dead *Salix*	MK163869
Cadophora sp.	Ascomycota	Ersaa	605-A	Native dead *Salix*	MK163892
Cadophora sp.	Ascomycota	Ersaa	605-C	Native dead *Salix*	MK163870
Cadophora sp.	Ascomycota	Iffiartarfik	588-A	Buried Arch	MK163791
Cadophora sp.	Ascomycota	Iffiartarfik	591-C	Buried Arch	MK163792
Cadophora sp.	Ascomycota	Iffiartarfik	592-B	Native dead *Salix*	MK163793
Cadophora sp.	Ascomycota	Kangeq	623-A	Exposed Hist	MK163802
Cadophora sp.	Ascomycota	Kangeq	623-B	Exposed Hist	MK163803
Cadophora sp.	Ascomycota	Qajaa	512-C	Buried Arch	MK163747
Cadophora sp.	Ascomycota	Garder (Igaliku)	325-A	Buried Arch	MK163700
Cadophora sp.	Ascomycota	Kangeq	219-A	Buried Arch	MN384790
Cadophora sp.	Ascomycota	Kangeq	219-B	Buried Arch	MN384791
Cadophora sp.	Ascomycota	Qajaa	414 02-D	Buried Arch	MK163748
Cadophora sp.	Ascomycota	Qajaa	508-A	Buried Arch	MN384792
Cadophora sp.	Ascomycota	Qoornoq	586-C	Native dead *Salix*	MK163811
Cadophora sp.	Ascomycota	Sermermiut	500-G	Exposed Arch	MK163779
Cephalotheca sp.	Ascomycota	Sermermiut	501-B	Exposed Arch	MK163784
Chaetomidium sp.	Ascomycota	Iffiartarfik	591-A	Buried Arch	MK163852
Chaetomidium sp.	Ascomycota	Iffiartarfik	591-B	Buried Arch	MK163890
Chaetomidium sp.	Ascomycota	Qoornoq	582-B	Buried Arch	MK163851
Chrysosporium sp.	Ascomycota	Qoornoq	580-C	Buried Arch	MK163849
Chrysosporium sp.	Ascomycota	Kilaarsafik (Sandnes)	43101-A	Buried Arch	MK163774
Chrysosporium sp.	Ascomycota	Kilaarsafik (Sandnes)	43101-B	Buried Arch	MK163775
Chrysosporium sp.	Ascomycota	Kilaarsafik (Sandnes)	43501-B	Buried Arch	MK163776
Cladosporium sp.	Ascomycota	Ersaa	604-A	Native dead *Salix*	MK163796
Cladosporium sp.	Ascomycota	Ersaa	604-D	Native dead *Salix*	MK163817
Cladosporium sp.	Ascomycota	Kangeq	615-C	Exposed Arch	MK163839
Cladosporium sp.	Ascomycota	Qoornoq	579-A	Buried Arch	MK163827
Cladosporium sp.	Ascomycota	Qoornoq	586-A	Native dead *Salix*	MK163881
Collarina sp.	Ascomycota	Garder (Igaliku)	329 02-A	Buried Arch	MK163701
Collophora sp.	Ascomycota	Austmannadal	570-B(a)	Native dead *Salix*	MK163863
Coniochaeta sp.	Ascomycota	Austmannadal	566-A	Exposed Hist	MK163823
Coniochaeta sp.	Ascomycota	Austmannadal	566-C	Exposed Hist	MK163788
Coniochaeta sp.	Ascomycota	Austmannadal	568-A	Native dead *Salix*	MK163876
Coniochaeta sp.	Ascomycota	Austmannadal	569-A	Native dead *Salix*	MK163789

Organism	Fungal type	Site	Isolate	Sample type	GenBank #
Coniochaeta sp.	Ascomycota	Austmannadal	566-B	Exposed Hist	MK163824
Cosmospora sp.	Ascomycota	Kangeq	609-D	Buried Arch	MK163818
Cosmospora sp.	Ascomycota	Qajaa	631-A	Buried Arch	MK163805
Cosmospora sp.	Ascomycota	Qajaa	631-B	Buried Arch	MK163806
Cosmospora sp.	Ascomycota	Nuulliit	521-D	Buried Arch	MK163736
Cosmospora sp.	Ascomycota	Nuulliit	521-E	Buried Arch	MK163737
Cosmospora sp.	Ascomycota	Qajaa	414 02-E	Buried Arch	MK163750
Cosmospora sp.	Ascomycota	Qajaa	512-D	Buried Arch	MK163749
Cytospora sp.	Ascomycota	Iffiartarfik	594-B	Native dead *Salix*	MK163794
Dacrymyces sp.	Basidiomycota	Austmannadal	30404-G	Exposed Hist	MK163874
Fomes sp.	Basidiomycota	Qoornoq	586-B	Native dead *Salix*	MK163828
Helotiales sp.	Ascomycota	Kangeq	300 04-B	Buried Arch	MK163714
Helotiales sp.	Ascomycota	Kangeq	306-D	Buried Arch	MK163715
Helotiales sp.	Ascomycota	Kangeq	619-E	Exposed Arch	MK163861
Helotiales sp.	Ascomycota	Qoornoq	579-B	Buried Arch	MK163847
Helotiales sp.	Ascomycota	Qoornoq	579-C	Buried Arch	MK163809
Helotiales sp.	Ascomycota	Kangeq	300 04-A	Buried Arch	MK163713
Hohenbuehelia sp.	Basidiomycota	Sermermiut	500-B	Exposed Arch	MK163787
Hymenoscyphus sp.	Ascomycota	Austmannadal	569-B	Native dead *Salix*	MK163877
Hypcreales sp.	Ascomycota	Kangeq	625-F	Exposed Hist	MK163821
Hypocrea sp.	Ascomycota	Kangeq	618-C	Exposed Arch	MK163841
Hypocrea sp.	Ascomycota	Kangeq	504-D	Exposed Arch	MK163716
Hypocrea sp.	Ascomycota	Kangeq	504-E	Exposed Arch	MK163717
Isaria sp.	Ascomycota	Qajaa	512-A	Buried Arch	MK163754
Lachnellula sp.	Ascomycota	Austmannadal	570-B(b)	Native dead *Salix*	MK163872
Lachnum sp.	Ascomycota	Ersaa	602-E	Native dead *Salix*	MK163856
Lecythophora sp.	Ascomycota	Sermermiut	500-A	Exposed Arch	MK163780
Lentinellus sp.	Basidiomycota	Iffiartarfik	594-D	Native dead *Salix*	MK163866
Lentinellus sp.	Basidiomycota	Iffiartarfik	595-A	Native dead *Salix*	MK163813
Lentinellus sp.	Basidiomycota	Iffiartarfik	595-B(a)	Native dead *Salix*	MK163795
Lentinellus sp.	Basidiomycota	Iffiartarfik	595-B(b)	Native dead *Salix*	MK163891
Lentinellus sp.	Basidiomycota	Sermermiut	501-D	Exposed Arch	MK163781
Leptodontidium sp.	Ascomycota	Austmannadal	304 04-D	Exposed Hist	MK163686
Leuconeurospora sp.	Ascomycota	Kangeq	625-E	Exposed Hist	MK163873
Metapochonia sp.	Ascomycota	Kangeq	625-C(a)	Exposed Hist	MK163804
Metapochonia sp.	Ascomycota	Kangeq	625-C(b)	Exposed Hist	MK163820
Microdochium sp.	Ascomycota	Ersaa	604-C	Native dead *Salix*	MK163842
Microdochium sp.	Ascomycota	Kangeq	615-B	Exposed Arch	MK163888
Microdochium sp.	Ascomycota	Kangeq	619-F	Exposed Arch	MK163836
Mortierella sp.	Mucoromycota	Ersaa	601-B	Buried Arch	MK163854
Mortierella sp.	Mucoromycota	Kangeq	609-B	Buried Arch	MK163831
Mortierella sp.	Mucoromycota	Sermermiut	556-A	Exposed Arch	MK163807
Mortierella sp.	Mucoromycota	Sermermiut	560-B	Exposed Arch	MK163822
Mortierella sp.	Mucoromycota	Kangeq	222 02-A	Buried Arch	MK163719

Organism	Fungal type	Site	Isolate	Sample type	GenBank #
Mortierella sp.	Mucoromycota	Qajaa	512-F	Buried Arch	MK163755
Mucor sp.	Mucoromycota	Austmannadal	567-C	Exposed Hist	MK163846
Mucor sp.	Mucoromycota	Kilaarsafik (Sandnes)	43601-A	Buried Arch	MK163777
Mucor sp.	Mucoromycota	Kilaarsafik (Sandnes)	43601-B	Buried Arch	MK163778
Mucor sp.	Mucoromycota	Sermermiut	500-D	Exposed Arch	MK163783
Mycena sp.	Basidiomycota	Qoornoq	583-A	Native dead *Salix*	MK163864
Mycena sp.	Basidiomycota	Qoornoq	583-D	Native dead *Salix*	MK163810
Oidiodendron sp.	Ascomycota	Kangeq	505-A	Exposed Arch	MK163720
Oidiodendron sp.	Ascomycota	Kangeq	505-B	Exposed Arch	MK163721
Oidiodendron sp.	Ascomycota	Kangeq	505-C	Exposed Arch	MK163722
Paraboeremia sp.	Ascomycota	Austmannadal	568-C	Native dead *Salix*	MK163826
Paraboeremia sp.	Ascomycota	Sermermiut	560-A	Exposed Arch	MK163825
Patinella sp.	Ascomycota	Garder (Igaliku)	321-A	Buried Arch	MK163702
Patinella sp.	Ascomycota	Kangeq	224-B	Buried Arch	MK163728
Patinella sp.	Ascomycota	Kangeq	305-A	Buried Arch	MK163723
Patinella sp.	Ascomycota	Kangeq	305-B	Buried Arch	MK163724
Patinella sp.	Ascomycota	Kangeq	306 02-A	Buried Arch	MK163727
Patinella sp.	Ascomycota	Kangeq	306-A	Buried Arch	MK163726
Patinella sp.	Ascomycota	Kangeq	306-B	Buried Arch	MK163729
Patinella sp.	Ascomycota	Kangerdluarssugssuaic	303 02-A	Buried Arch	MK163692
Patinella sp.	Ascomycota	Kangerdluarssugssuaic	303 02-B	Buried Arch	MK163693
Patinella sp.	Ascomycota	Kangerdluarssugssuaic	303 02-C	Buried Arch	MK163694
Patinella sp.	Ascomycota	Kangerdluarssugssuaic	310 04-C	Buried Arch	MK163696
Patinella sp.	Ascomycota	Kangerdluarssugssuaic	439-A	Buried Arch	MK163697
Patinella sp.	Ascomycota	Kangerdluarssugssuaic	439-B	Buried Arch	MK163698
Patinella sp.	Ascomycota	Kangerdluarssugssuaic	439-C	Buried Arch	MK163699
Patinella sp.	Ascomycota	Nuulliit	521-A	Buried Arch	MK163739
Patinella sp.	Ascomycota	Nuulliit	521-B	Buried Arch	MK163740
Patinella sp.	Ascomycota	Nuulliit	521-C	Buried Arch	MK163741
Patinella sp.	Ascomycota	Nuulliit	521-F	Buried Arch	MK163742
Patinella sp.	Ascomycota	Qajaa	414 02-A	Buried Arch	MK163757
Patinella sp.	Ascomycota	Qajaa	414 02-B	Buried Arch	MK163758
Patinella sp.	Ascomycota	Qajaa	414 02-C1	Buried Arch	MK163759
Patinella sp.	Ascomycota	Qajaa	414-A	Buried Arch	MK163760
Patinella sp.	Ascomycota	Qajaa	414-E	Buried Arch	MK163761
Patinella sp.	Ascomycota	Qajaa	508-C	Buried Arch	MK163756
Penicillium sp.	Ascomycota	Austmannadal	567-A	Exposed Hist	MK163808
Penicillium sp.	Ascomycota	Austmannadal	567-B	Exposed Hist	MK163840
Penicillium sp.	Ascomycota	Ersaa	602-A	Native dead *Salix*	MK163814
Penicillium sp.	Ascomycota	Ersaa	602-B	Native dead *Salix*	MK163868
Penicillium sp.	Ascomycota	Ersaa	605-B	Native dead *Salix*	MK163798
Penicillium sp.	Ascomycota	Iffiartarfik	590-A	Buried Arch	MK163830
Penicillium sp.	Ascomycota	Iffiartarfik	590-C	Buried Arch	MK163865
Penicillium sp.	Ascomycota	Iffiartarfik	594-A	Native dead *Salix*	MK163812

Organism	Fungal type	Site	Isolate	Sample type	GenBank #
Penicillium sp.	Ascomycota	Kangeq	615-A (a)	Exposed Arch	MK163871
Penicillium sp.	Ascomycota	Kangeq	615-A (b)	Exposed Arch	MK163882
Penicillium sp.	Ascomycota	Kangeq	619-A	Exposed Arch	MK163834
Penicillium sp.	Ascomycota	Kangeq	619-B	Exposed Arch	MK163887
Penicillium sp.	Ascomycota	Kangeq	619-C	Exposed Arch	MK163886
Penicillium sp.	Ascomycota	Kangeq	619-D	Exposed Arch	MK163835
Penicillium sp.	Ascomycota	Kangeq	622-A	Exposed Hist	MK163837
Penicillium sp.	Ascomycota	Kangeq	625-A	Exposed Hist	MK163819
Penicillium sp.	Ascomycota	Kangeq	625-B	Exposed Hist	MK163838
Penicillium sp.	Ascomycota	Qoornoq	583-B	Native dead *Salix*	MK163875
Penicillium sp.	Ascomycota	Qoornoq	583-E	Native dead *Salix*	MK163880
Penicillium sp.	Ascomycota	Sermermiut	500-F	Exposed Arch	MK163785
Phaeosphaeria sp.	Ascomycota	Ersaa	604-B	Native dead *Salix*	MK163816
Phialemonium sp.	Ascomycota	Kangeq	610-C	Buried Arch	MK163833
Phialemonium sp.	Ascomycota	Kangeq	504-A	Exposed Arch	MK163730
Phialemonium sp.	Ascomycota	Kangeq	504-B	Exposed Arch	MK163731
Phialemonium sp.	Ascomycota	Kangeq	504-C	Exposed Arch	MK163732
Phialemonium sp.	Ascomycota	Kangeq	507-C	Exposed Arch	MK163733
Phialemonium sp.	Ascomycota	Qajaa	414-B	Buried Arch	MK163765
Phialemonium sp.	Ascomycota	Qajaa	414-C	Buried Arch	MK163766
Phialemonium sp.	Ascomycota	Qajaa	414-D	Buried Arch	MK163767
Phialemonium sp.	Ascomycota	Qajaa	511-A	Buried Arch	MK163762
Phialemonium sp.	Ascomycota	Qajaa	511-D	Buried Arch	MK163763
Phialemonium sp.	Ascomycota	Qajaa	512-B	Buried Arch	MK163764
Phialemonium sp.	Ascomycota	Qajaa	514-A	Buried Arch	MK163768
Phialemonium sp.	Ascomycota	Qajaa	514-B	Buried Arch	MK163769
Phialemonium sp.	Ascomycota	Sermermiut	501-C(a)	Exposed Arch	MK163782
Phialemonium sp.	Mucoromycota	Sermermiut	501-C(b)	Exposed Arch	MK163786
Phialophora sp.	Ascomycota	Qajaa	508-B	Buried Arch	MK163773
Phoma sp.	Ascomycota	Kangeq	507-D	Exposed Arch	MK163734
Pseudeurotium sp.	Ascomycota	Austmannadal	30404-E	Exposed Hist	MK163843
Pseudeurotium sp.	Ascomycota	Kangeq	610-B	Buried Arch	MK163859
Pseudeurotium sp.	Ascomycota	Kangeq	224-A	Buried Arch	MK163735
Pseudeurotium sp.	Ascomycota	Kangeq	609-A	Buried Arch	MK163799
Pseudeurotium sp.	Ascomycota	Kangeq	609-C	Buried Arch	MK163832
Pseudeurotium sp.	Ascomycota	Kangeq	609-E	Buried Arch	MK163800
Pseudeurotium sp.	Ascomycota	Kangeq	610-A	Buried Arch	MK163858
Pseudeurotium sp.	Ascomycota	Nuulliit	521-G	Buried Arch	MK163743
Pseudeurotium sp.	Ascomycota	Nuulliit	521-H	Buried Arch	MK163746
Pseudeurotium sp.	Ascomycota	Nuulliit	523 03-A	Buried Arch	MK163744
Pseudeurotium sp.	Ascomycota	Nuulliit	523 03-B	Buried Arch	MK163745
Pseudeurotium sp.	Ascomycota	Qajaa	511-K	Buried Arch	MK163770
Pseudeurotium sp.	Ascomycota	Qajaa	511-L	Buried Arch	MK163771
Pseudogymnoascus sp.	Ascomycota	Qoornoq	580-A	Buried Arch	MK163848

**Organism**	**Fungal type**	**Site**	**Isolate**	**Sample type**	**GenBank #**
Pseudogymnoascus sp.	Ascomycota	Qoornoq	582-C	Buried Arch	MK163878
Pseudogymnoascus sp.	Ascomycota	Qoornoq	583-C	Native dead *Salix*	MK163879
Tetracladium sp.	Ascomycota	Iffiartarfik	591-G	Buried Arch	MK163853
Tetracladium sp.	Ascomycota	Kangeq	615-D	Exposed Arch	MK163860
Tetracladium sp.	Ascomycota	Qoornoq	580-D	Buried Arch	MK163850
Tolypocladium sp.	Ascomycota	Ersaa	602-C	Native dead *Salix*	MK163815
Tolypocladium sp.	Ascomycota	Ersaa	602-F	Native dead *Salix*	MK163889
Tolypocladium sp.	Ascomycota	Kangeq	618-A	Exposed Arch	MK163801
Tolypocladium sp.	Ascomycota	Kangeq	618-B	Exposed Arch	MK163883
Tremellales sp.	Basidiomycota	Qajaa	511 01-J	Buried Arch	MK163753
Tremellales sp.	Basidiomycota	Qajaa	511-G	Buried Arch	MK163751
Tremellales sp.	Basidiomycota	Qajaa	511-I	Buried Arch	MK163752
Tympanis sp.	Ascomycota	Ersaa	602-D	Native dead *Salix*	MK163855
Umbelopsis sp.	Mucoromycota	Austmannadal	304 04-A	Exposed Hist	MK163689
Umbelopsis sp.	Mucoromycota	Austmannadal	304 04-B	Exposed Hist	MK163687
Umbelopsis sp.	Mucoromycota	Austmannadal	304 04-C	Exposed Hist	MK163688
Umbelopsis sp.	Mucoromycota	Qajaa	508-D	Buried Arch	MK163772
Wettsteinina sp.	Ascomycota	Iffiartarfik	588-B	Buried Arch	MK163829
Xenopolyscytalum sp.	Ascomycota	Kangeq	305-C	Buried Arch	MK163725
Xenopolyscytalum sp.	Ascomycota	Kangeq	306-C	Buried Arch	MK163706
Xenopolyscytalum sp.	Ascomycota	Kangeq	507-F	Exposed Arch	MK163718
Xenopolyscytalum sp.	Ascomycota	Kangeq	608-D	Buried Arch	MK163857
Xenopolyscytalum sp.	Ascomycota	Kangeq	219-D	Buried Arch	MK163710
Xenopolyscytalum sp.	Ascomycota	Kangeq	219-E	Buried Arch	MK163703
Xenopolyscytalum sp.	Ascomycota	Kangeq	222 02-B	Buried Arch	MK163704
Xenopolyscytalum sp.	Ascomycota	Kangeq	222 02-C	Buried Arch	MK163705
Xenopolyscytalum sp.	Ascomycota	Kangeq	306 02-D	Buried Arch	MK163711
Xenopolyscytalum sp.	Ascomycota	Kangeq	306 02-E	Buried Arch	MK163712
Xenopolyscytalum sp.	Ascomycota	Kangeq	507-A	Exposed Arch	MK163707
Xenopolyscytalum sp.	Ascomycota	Kangeq	507-B	Exposed Arch	MK163708
Xenopolyscytalum sp.	Ascomycota	Kangeq	507-E	Exposed Arch	MK163709
Xenopolyscytalum sp.	Ascomycota	Kangerdluarssugssuaic	307-B	Buried Arch	MK163690
Xenopolyscytalum sp.	Ascomycota	Kangerdluarssugssuaic	310 04-B	Buried Arch	MK163695
Xenopolyscytalum sp.	Ascomycota	Kangerdluarssugssuaic	310-B	Buried Arch	MK163691
Xenopolyscytalum sp.	Ascomycota	Qajaa	631-C	Buried Arch	MK163862
Xenopolyscytalum sp.	Ascomycota	Sermermiut	556-C	Exposed Arch	MK163844
Xenopolyscytalum sp.	Ascomycota	Sermermiut	559-B	Exposed Arch	MK163845

Supplementary Figure S5

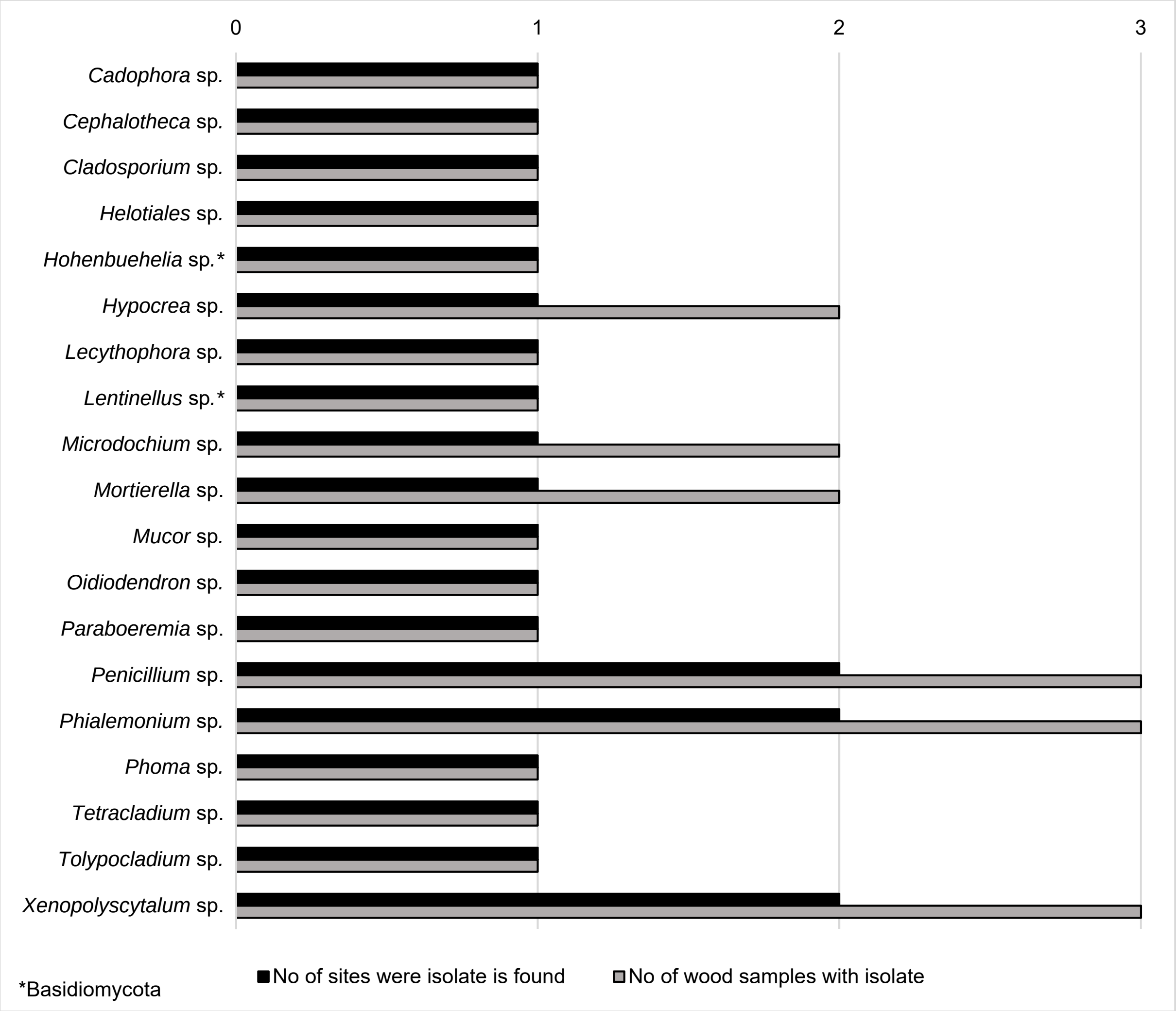

Supplementary Figure S5: Identified filamentous fungal isolates grown from exposed archaeological wood samples collected at three archaeological sites. The figure specify the number of unique wood samples from where the filamentous fungal isolates were grown and the number of sites where the isolates were found.

Supplementary Figure S6

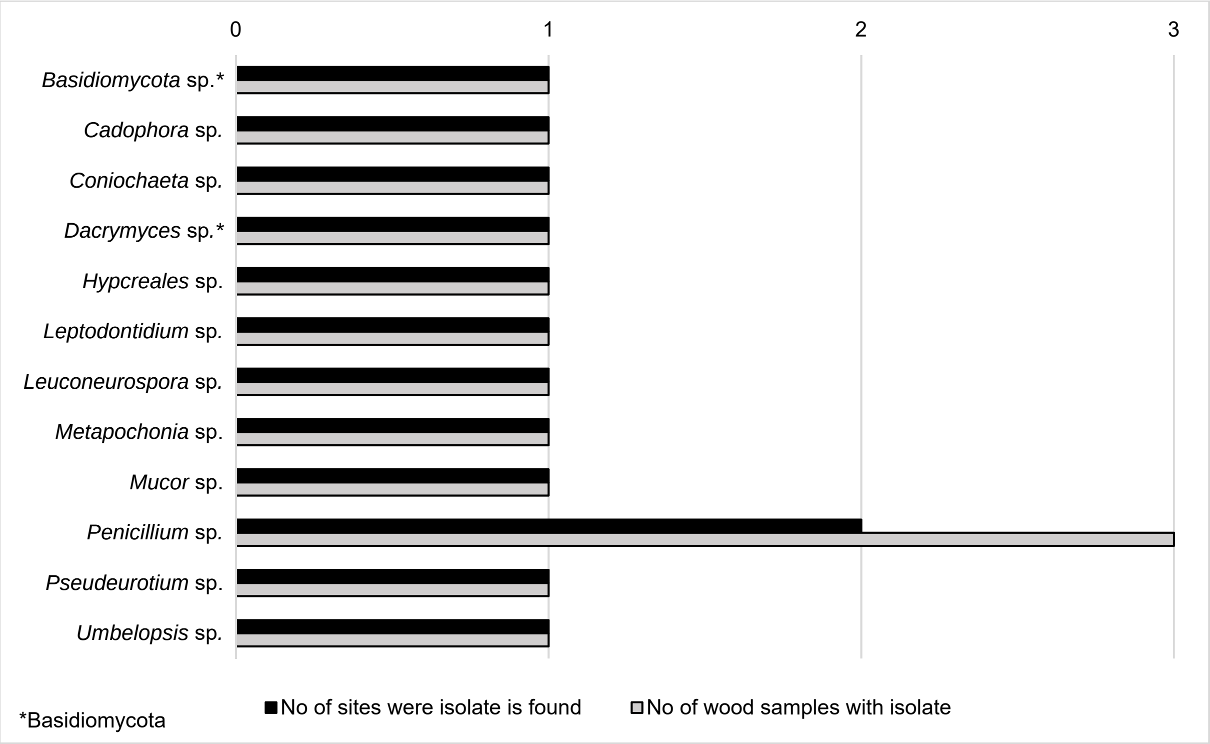

Supplementary Figure S6: Identified filamentous fungal isolates grown from exposed historical wood samples collected at two archaeological sites. The figure specify the number of unique wood samples from where the filamentous fungal isolates were grown and the number of sites where the isolates were found.

Supplementary Figure S7

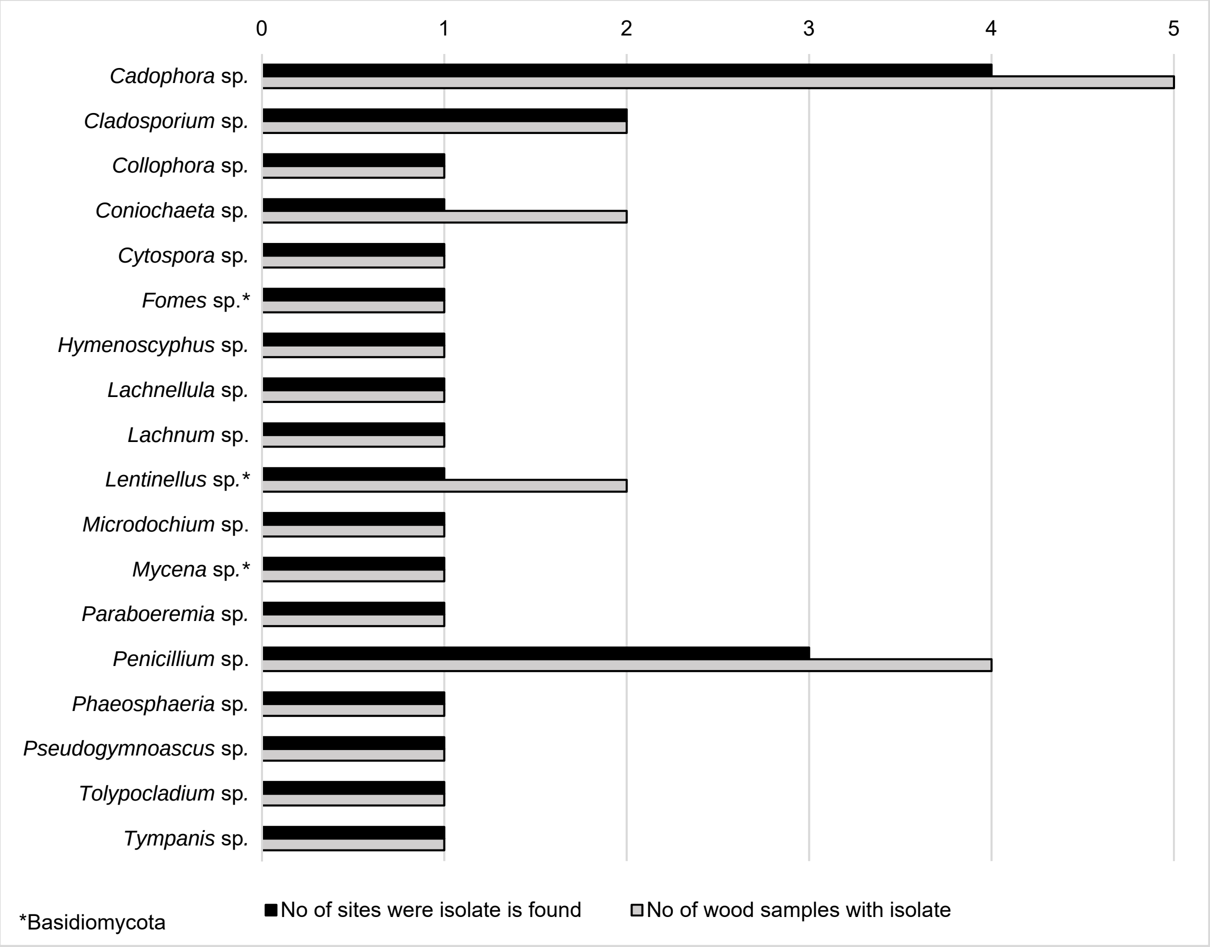

Supplementary Figure S7: Identified filamentous fungal isolates grown from native dead *Salix glauca* collected at four archaeological sites. The figure specify the number of unique wood samples from where the filamentous fungal isolates were grown and the number of sites where the isolates were found.

Supplementary Table S8

Supplementary Table S8: Summary table of identified Basidiomycota in extracted gDNA

**Genera/species**	**Site**	**No. of identified strains in sample**	**Type of wood sample**
Galerina marginata	Kangeq	2	Exposed Historical
Hymenochaete spreta	Austmannadal	3	Exposed Historical
Hyphoderma medioburiense	Iffiartarfik	1	Native dead Salix
Hyphoderma obtusiforme	Austmannadal	2	Exposed Historical
Lentinellus omphalodes	Iffiartarfik	3	Native dead Salix
Lentinellus omphalodes	Qoornoq	3	Native dead Salix
Lentinellus vulpinus	Sermermiut	1	Exposed Archaeological
Lepista densifolia	Sermermiut	1	Exposed Archaeological
Lyomyces sp.	Nuulliit	1	Buried Archaeological
Mycena alexandri	Ersaa	3	Native dead Salix
Mycena alnetorum	Ersaa	1	Native dead Salix
Mycena alnetorum	Qoornoq	3	Native dead Salix
Mycena leptocephala	Sermermiut	1	Exposed Archaeological
Peniophorella praetermissa	Austmannadal	2	Exposed Historical
Sphaerobolus stellatus	Austmannadal	1	Exposed Historical
Vuilleminia macrospora	Austmannadal	1	Exposed Historical
Uncultured fungus isolate	Kangeq	1	Exposed Archaeological

Supplementary Table S9

Supplementary Table S9: Detailed information on species identification of Basidiomycota in extracted gDNA, inkluding sequences and best Blast in GenBank

Sample no.	Site	Sample type	BLAST type	Sequence	Best BLAST	Max score	Total score	Query cover	E value	Ident	Accession
304 04 I	Austmannadal	Exp Hist	De Novo Assemble	GACTACAACTCGGACAGCCAAAGACTGCCAGATTTTAAATTTGAGCT<br>TTTCCCGCTTCACTCGCAGTTACTAGGGGAATCCTTGTTAGTTTCTTTT<br>CCTCCGCTTATTGATATGCTTAAGTTCAGCGGGTAGTCCTGCCTGATT<br>TGAGGTCAAATGCAAAAAGATTTGTTCCACTATGGAAACAATTTGAA<br>GCACGAACACATAAATCTCATCAAAGATTCGGCAGCGCAGATAATTA<br>TTACACTGGAGAACACTTGATAAATATTCGCGCTAATGCATTTGAGA<br>AGAGCTGAAAGTATGTTTCCAGCAGCTTCCAATATCCAGTACCAGCC<br>GTCAGAAAAATCTGAGGGGACTGAGAATAACATGACACTCAAACAG<br>GCATGCTCCTCGGAATACCAAGGAGCGCAAGGTGCGTTCAAAGATTC<br>GATGATTCACT	*Hyphoderma obtusiforme*	540	540	71%	1.00E-149	98%	JN572909.1
304 04 II	Austmannadal	Exp Hist	De Novo Assemble	TACAACTCGGACGATTGAAAACCGCCAGATTACAAATTTGAGCTCTT<br>CCCGCTTCACTCGCCGTTACTAGGGGAATCCTTGTTAGTTTCTTTTCCT<br>CCGCTTATTGATATGCTTAAGTTCAGCGGGTAGTCCTACCTGATTTGA<br>GGTCAGATGACATGAAGTTATTGTCTCAGTCAAGAGACTATTAGAAG<br>CAGTCACTTCAAGTTTATAACAACAGCGCTACAAATCAACAATGAAA<br>TTGTTTCTTATCACACCATTATGAGTTATAAACCGCGGCGGACCAGCT<br>AATGCATTTCGAGACGAGCTGCTATARTAAAATAGCAGCAACATCCA<br>AGTCCAAACTATAACCTTTCTTCAATAAAGAAAAAAGGTTAAGTTTG<br>AGAGTATTCACGACACTCAAACAGGCATGCTCCTCGGAATACCAAGG<br>GGCGCAAGATGCGTTCAAAGATTCGATGATTCAC	*Sphaerobolus stellatus*	651	651	77%	0	99%	KM586165.1
304 04 III	Austmannadal	Exp Hist	De Novo Assemble	GACTACAACTCGGACAGCCAAAGACTGCCAGATTTTAAATTTGAGCT<br>TTTCCCGCTTCACTCGCAGTTACTAGGGGAATCCTTGTTAGTTTCTTTT<br>CCTCCGCTTATTGATATGCTTAAGTTCAGCGGGTAGTCCTGCCTGATT<br>TGAGGTCAAATGCAAAAAGATTTGTTCCACTATGGAAACGATTTGAA<br>GCACGAACACATAAATCTCATCAAAGATTCGGCAGCGCAGATAATTA<br>TTACACTGGAGAACACTTGATAAATATTCGCGCTAATGCATTTGAGA<br>AGAGCTGAAAGTATGTTTCCAGCAGCTTCCAATATCCAGTACCAGCC<br>GTCAGAAAAATCTGAGGGGACTGAGAATAACATGACACTCAAACAG<br>GCATGCTCCTCGGAATACCAAGGAGCGCAAGGTGCGTTCAAAGATTC<br>GAT	*Hyphoderma obtusiforme*	531	531	70%	7.00E-147	98%	JN572909.1

Sample no.	Site	Sample type	BLAST type	Sequence	Best BLAST	Max score	Total score	Query cover	E value	Ident	Accession
500 01 I	Semermuit	Exp Arch	De Novo Assemble	TTACAACTCGGACGGCCACAGGGACCGCCAGATTTTAAATTTGAGCT<br>TTTCCCGCTTCACTCGCAGTTACTAGGGGAATCCTTGTTAGTTTCTTTT<br>CCTCCGCTTATTGATATGCTTAAGTTCAGCGGGTAGTCCTACCTGATT<br>TGAGGTCAAATAGTCAATTAAATTGTCCCTTGCGAGACGGTTATAAG<br>CAGGTTCCCATAATTTTGCTTCGCAGTCAAATGGCATAGATAATTATC<br>ACACCAAGTGACGGTCCACAAGAGATCCCACTAATGCATTTAAAGGG<br>AGCAGACCATCCACTGAAGGAAGCCAGCAAGCCCTCACATCCAAGCC<br>TCGCTCAAGCTCGTAAAAGCGAACAAGGTTGATAATTTAATGACACT<br>CAAACAGGCATGCCCTTCGGAATACCAAAGGGCGCAAGGTGCGTTC<br>AAAGATTCGATGATTCAC	*Mycena*	821	821	100%	0	100%	JF519506.1
					Mycena leptocephala	809	809	100%	0	99%	HQ604773.1
501 01 I	Semermuit	Exp Arch	De Novo Assemble	GATTACAACTCGGACGGCCGAAGACCGCCAGATTACAAATTTGAGCT<br>TTTCCCGCTTCACTCGCAGTTACTAGGGGAATCCTTGTTAGTTTCTTTT<br>CCTCCGCTTATTGATATGCTTAAGTTCAGCGGGTAGTCCCGCCTGATC<br>TGAGGTCAAGTTTCGATGAAAGTGTCCCTTGCGAGACGGTTGGAAG<br>CGGGTCCCCCACGAGAGCTAAACCCACGGCGTAGACAATTATCACAC<br>CGAGGCCGCAAAGGGTCCTGCTAATGCCTTTGAGAGGAGCCGATCC<br>CACCGCGAGGGTGGGGGTCCGGCAAAGCCTCCAAGTCCAAGCCCCG<br>ACACCCCTCGCAAAAGGGGGTGGGGTTGAGAATTTCACGACACTCA<br>GACAGGCGTACCCCTCGGAATACCAAGGGGTGCAAGGTGCGTTCAA<br>AGATTCGATGATTCACTGAATTCTGCA	*Lentinellus vulpinus*	667	667	100%	0	93%	AF347097.1
501 01 III	Semermuit	Exp Arch	De Novo Assemble	CTCTAGATTACAACTCGGACGGCCAAAGACCGCCAGATTTTAAATTT<br>GAGCTTTTCCCGCTTCACTCGCAGTTACTAGGGGAATCCTTGTTAGTT<br>TCTTTTCCTCCGCTTATTGATATGCTTAAGTTCAGCGGGTAGTCCTAC<br>CTGATTTGAGGTCAAAATGTCAGAATTTGTCCAAGTCAATGGACGGT<br>TAGAAGCTGAACCCCATGTAAAACTGCTTCACGGCAATGGCGTAGAT<br>AATTATCACACCAGAGCTGGTCCACAAAGGTTCCGCTAATGCATTTA<br>AGAGGAGCCGACTTCTGAGAAGCCCGCAACCCCCACATCCAAGCCA<br>AATTCAACTCGCAAAAGCTGAAAAGGTTGAGAATTTAATGACACTCA<br>AACAGGCATGCTCCTCGGAATACCAAGGAGCGCAAGGTGCGTTCAA<br>AGATTCGATGATTCACTGAATTCTGC	*Lepista densifolia*	774	774	100%	0	98%	KJ681001.1
523 01 II	Nuulliit	Buried Arch	De Novo Assemble	GCAGAATTCAGTGAATCATCGAATCTTTGAACGCACCTTGCACTCCTT<br>GGTATTCCGAGGAGTATGCCTGTTTGAGTGTCTGACTTTCATCAATCC<br>TCCTTTACTTTTGTAATGGTTGGCTTGGATTTGGGGATTGCTGGGCCT<br>TGTGTCTGGCTTCCCTTGAATGCATTAGTTGAATTGACTTGCAACGCT<br>ATGTGTGGTGTGATAATATCTTCACTGCATAGTTGTAATGAAGTTTG<br>GCTTCTAACCGTCTTTCATTAGACAATCTTTGACCATTAGACCTCAAA<br>TCAGGTAGGACTACCCGCTGAACTTAAGCATATCAATAAGCGGAGG<br>AAAAGAAACTAACAAGGATTCCCCTAGTAACTGCGAGTGAAGCGGG<br>AAAAGCTCAAATTTAAAATCTGGTAGCTTCGCTGCCCGAGTTGTAAT<br>C	*Lyomyces* sp.	597	597	76%	7.00E-167	99%	JX857750.1

Sample no.	Site	Sample type	BLAST type	Sequence	Best BLAST	Max score	Total score	Query cover	E value	Ident	Accession
568 01 I	Austmannadal	Exp Hist	De Novo Assemble	TTGCAGAATTCAGTGAATCATCGAATCTTTGAACGCACCTTGCGCCCTTTGGTATTCCGAAGGGCATGCCTGTTTGAGTGTCATGTTAATCTCAATCCCCTTGTTTCTTAATTGAGGCTCGGAGGTTGGACTTGGAGGTTTGCTGGCCCCTCTTGTGGTCGGCTCCTCTTAAATGCATTAGCTGGACTTTGGTTCTGCGTGAAGGTGTAATAGTTTATTCGCCATTAGCGCTTGCCTAACGAGTCTGCTTCTAATCGTCTTCGGACAAGGATTAAGTTCCTTCTTGACTCTTTGACCTCAAATCAGGTAGGACTACCCGCTGAACTTAAGCATATCAATAAGCGGAGGAAAAGAAACTAACAAGGATTCCCCTAGTAACTGCGAGTGAAGCGGGAAAAGCTCAAATTTAAAATCTGGCGTCCTTTGGGCGTCCGAGTTGTAATC	*Hymenochaete spreta*	623	623	83%	1.00E-174	97%	AY558597.1
568 01 II	Austmannadal	Exp Hist	De Novo Assemble	TTACAACTCGGACGCCCAAAGGACGCCAGATTTTAAATTTGAGCTTTTCCCGCTTCACTCGCAGTTACTAGGGGAATCCTTGTTAGTTTCTTTTCCTCCGCTTATTGATATGCTTAAGTTCAGCGGGTAGTCCTACCTGATTTGAGGTCAAAGAGTCAAGAAGGAACTTAATCCTTGTCCGAAGACGATTAGAAGCAGACTCGTTAGGCAAGCGCTAATGGCGAATAAACTATTACACCTTCACGCAGAACCAAAGTCCAGCTAATGCATTTAAGAGGAGCCGACCACAAGAGGGGCCAGCAAACCTCCAAGTCCAACCTCCGAGCCTCAATTAAGAAACAAGGGGATTGAGATTAACATGACACTCAAACAGGCATGCCCTTCGGAATACCAAAGGGCGCAAGGTGCGTTCAAAGATTCGATGATTCACTGAATTCTGCAA	*Hymenochaete spreta*	623	623	83%	1.00E-174	97%	AY558597.1
568 01 III	Austmannadal	Exp Hist	De Novo Assemble	TTCTCCAGATTACAACTCGGACGCCCAAAGGACGCCAGATTTTAAATTTGAGCTTTTCCCGCTTCACTCGCAGTTACTAGGGGAATCCTTGTTAGTTTCTTTTCCTCCGCTTATTGATATGCTTAAGTTCAGCGGGTAGTCCTACCTGATTTGAGGTCAAAGAGTCAAGAAGGAACTTAATCCTTGTCCGAAGACGATTAGAAGCAGACTCGTTAGGCAAGCGCTAATGGCGAATAAACTATTACACCTTCACGCAGAACCAAAGTCCAGCTAATGCATTTAAGAGGAGCCGACCACAAGAGGGGCCAGCAAACCTCCAAGTCCAACCTCCGAGCCTCAATTAAGAAACAAGGGGATTGAGATTAACATGACACTCAAACAGGCATGCCCTTCGGAATACCAAAGGGCGCAAGGTGCGTTCAAAGATTCGATGATTCACTGAATTCTGC	*Hymenochaete spreta*	619	619	81%	2.00E-173	97%	AY558597.1
570 01 I	Austmannadal	Exp Hist	Forward	TTACTAGGGGAATCCTTGTTAGTTTCTTTTCCTCCGCTTATTGATATGCTTAAGTTCAGCGGGTAGTCCTACCTGATTTGAGGTCAAAGATTGAAAGATTGTCCAGAGGACAGTTAGAAGCCAGACCCATAGATATTCCACAGACAGTACAAAGCGTAGATAATTATCACGCCATGAAGCCTTAGGAACAAGTCCAAGCTAATGCATTCAAGGGAAGCCAGACCGAATCAAAACGGCCAGCAAGCCCCCAAGTCCAAGCCACCAGTCAAATTTAAAATCCAACTGGGGTTGATGATTTCAAGACACTCAAACAGGCATGCTCCTAGGAATAGCCCAGGAGCGCAAGGTGCGTTCAAAGATTCGATGATTCACTGAATTCTGCAATTCACATTACTTATCGCATTTCGCTGCGTTCTTCATCGAA	*Peniophorella praetermissa*	739	739	99%	0	98%	JQ358804.1
			Reverse	-							

Sample no.	Site	Sample type	BLAST type	Sequence	Best BLAST	Max score	Total score	Query cover	E value	Ident	Accession
570 01 II	Austmannadal	Exp Hist	De Novo Assemble	ACTCGGACGGCAGAACCGCCAGATTTTAAATTTGAGCTCTTCCCGCTTCACTCGCAGTTACTAGGGGAATCCTTGTTAGTTTCTTTTCCTCCGCTTATTGATATGCTTAAGTTCAGCGGGTAGTCCTACCTGATTTGAGGTCAAAGATTGAAAGATTGTCCAGAGGACAGTTAGAAGCCAGACCCATAGATATTCCACAGACAGTACAAAGCGTAGATAATTATCACGCCATGAAGCCTTAGGAACAAGTCCAAGCTAATGCATTCAAGGGAAGCCAGACCGAATCAAAACGGCCAGCAAGCCCCCAAGTCCAAGCCACCAGTCAAATTTAAAATCCAACTGGGGTTGATGATTTCAAGACACTCAAACAGGCATGCTCCTAGGAATAGCCCAGGAGCGCAAGGTGCGTTCAAAGATTCGATGATTCAC	*Peniophorella praetermissa*	750	750	100%	0	98%	JQ358804.1
570 01 III	Austmannadal	Exp Hist	De Novo Assemble	TCTAGATTACAACTCGGACGGCCAAAGACCGCCAGATTTTAAATTTGAGCTTTTCCCGCTTCACTCGCAGTTACTAGGGGAATCCTTGTTAGTTTCTTTTCCTCCGCTTATTGATATGCTTAAGTTCAGCGGGTAGTCCTACCTGATTTGAGGTCAGATTCAAAGGTGAATTGTCCGAAGACGGTTAGAAGCGGGATCCGGAGTAGTAGGTGACGGCGCAGATAATTATCACACCGACCGTTCCTCCGAGCTAAAGCCCAGCTAATGCATTTGAGAGGAGCCGACCGTCGCCAGCAATGCCTCCAAGTCCAAGCCACAGCAAGCGAACAAACGCTTGCGGGTTGATAGATTCACGACGCTCAAACAGGCATGCTCCTCGGAATACCAAGGAGCGCAAGGTGCGTTCAAAGATTCGATGATTCACTGAATTC	*Vuilleminia macrospora*	671	671	100%	0	95%	HM046885.1
583 01 I	Qoornoq	Native dead Salix	De Novo Assemble	ACAACTCGGACGGCTACAAGAACCGCCAGATTTTAAATTTGAGCTTTTCCCGCTTCACTCGCAGTTACTAGGGGAATCCTTGTTAGTTTCTTTTCCTCCGCTTATTGATATGCTTAAGTTCAGCGGGTAGTCCTACCTGATTTGAGGTCAAAATGTCAAAAGTTGTCCGAAGACGGTTATGAGCGGGTTCCCATAAGTTTTGCTTCAAGGTCAAATGGCATAGATAATTATCACACCAAGTGACGGTCCACAAGAGATCCCACTAATGCATTTAAAGGGAGCAGACCATCCACTGAAGGAAGCCAGCAAGCCCTCACATCCAAGCCTAACTCAAGCCGGTAAAAGCGAGCAAGGTTGATAATTTAATGACACTCAAACAGGCATGCCCTTCGGAATACCAAAGGGCGCAAGGTGCGTTCAAAGATTCGA	*Mycena*	784	784	100%	0	99%	KF476767.1
					Mycena sp.	782	782	100%	0	99%	JF908495.1
					Mycena alnetorum	778	778	98%	0	99%	JF908426.1

Sample no.	Site	Sample type	BLAST type	Sequence	Best BLAST	Max score	Total score	Query cover	E value	Ident	Accession
583 01 II	Qoornoq	Native dead Salix	Forward	CACTCGGACGGCTACAAGAACCGCCAGATTTTAAATTTGAGCTTTTCCCGCTTCACTCGCAGTTACTAGGGGAATCCTTGTTAGTTTCTTTTCCTCCGCTTATTGATATGCTTAAGTTCAGCGGGTAGTCCTACCTGATTTGAGGTCAAAATGTCAAAAGTTGTCCGAAGACGGTTATGAGCGGGTTCCCATAAGTTTTGCTTCAAGGTCAAATGGCATAGATAATTATCACACCAAGTGACGGTCCACAAGAGATCCCACTAATGCATTTAAAGGGAGCAGACCATCCACTGAAGGAAGCCAGCAAGCCCTCACATCCAAGCCTAACTCAAGCCGGTAAAAGCGAGCAAGGTTGATAATTTAATGACACTCAAACAGGCATGCCCTTCGGAATACCAAAGGGCGCAAGGTGCGTTCAAAGATTCGATGATTCACTGAATTCTGCAATTCACATTACTTATCGCATTTCGCTGCTT	*Mycena alnetorum*	865	865	98%	0	99%	JF908426.1
			Reverse	-							
583 01 III	Qoornoq	Native dead Salix	De Novo Assemble	GCAGAATTCAGTGAATCATCGAATCTTTGAACGCACCTTGCGCCCTTTGGTATTCCGAAGGGCATGCCTGTTTGAGTGTCATTAAATTATCAACCTTGCTCGCTTTTACCGGCTTGAGTTAGGCTTGGATGTGAGGGCTTGCTGGCTTCCTTCAGTGGATGGTCTGCTCCCTTTAAATGCATTAGTGGGATCTCTTGTGGACCGTCACTTGGTGTGATAATTATCTATGCCATTTGACCTTGAAGCAAAACTTATGGGAACCCGCTCATAACCGTCTTCGGACAACTTTTGACATTTTGACCTCAAATCAGGTAGGACTACCCGCTGAACTTAAGCATATCAATAAGCGGAGGAAAAGAAACTAACAAGGATTCCCCTAGTAACTGCGAGTGAAGCGGGAAAAGCTCAAATTTAAAATCTGGCGGTTCTTGTAGCCGTCCGAGTTGTA	*Mycena* sp.	817	817	100%	0	99%	JF908495.1
					Mycena alnetorum	811	811	98%	0	99%	JF908426.1
					Mycena aff. murina	806	806	100%	0	99%	AF335444.1
584 01 I	Qoornoq	Native dead Salix	De Novo Assemble	TTGCAGAATTCAGTGAATCATCGAATCTTTGAACGCACCTTGCACCCCTTGGTATTCCGAGGGGTACGCCTGTTTGAGTGTCGTGAAATTCTCAACCCCGCCCCCTTTTGCGAGGGGCGTCGGTGGCTTGGACTTGGAGGCTTTTGCCGGGAAARGGTTTCGACCCACTTCTCGGCTCCTCTCGAAGGCATTAGTAGGACCCTTTGCGGCCTCGGTGTGATAATTGTCTACGCCGTGGGTTTAGCATGTCATGGGACCCGCTTCCAACCGTCTCGCAAGGGACACTTTCAATCGAAACTTGACCTCAGATCAGGCGGGACTACCCGCTGAACTTAAGCATATCAATAAGCGGAGGAAAAGAAACTAACAAGGATTCCCCTAGTAACTGCGAGTGAAGCGGGAAAAGCTCAAATTTGTAATCTGGCGGCCTTCGGTCGTCCGAGTTGTAA	*Lentinellus omphalodes*	780	780	100%	0	98%	AF506418.1

Sample no.	Site	Sample type	BLAST type	Sequence	Best BLAST	Max score	Total score	Query cover	E value	Ident	Accession
584 01 II	Qoornoq	Native dead Salix	De Novo Assemble	TTGCAGAATTCAGTGAATCATCGAATCTTTGAACGCACCTTGCACCCC<br>TTGGTATTCCGAGGGGTACGCCTGTTTGAGTGTCGTGAAATTCTCAA<br>CCCCGCCCCCTTTTGCGAGGGGCGTCGGTGGCTTGGACTTGGAGGCT<br>TTTGCCGGGAAAGGGTTTCGACCCACTTCTCGGCTCCTCTCGAAGGC<br>ATTAGTAGGACCCTTTGCGGCCTCGGTGTGATAATTGTCTACGCCGT<br>GGGTTTAGCATGTCATGGGACCCGCTTCCAACCGTCTCGCAAGGGAC<br>ACTTTCAATCGAAACTTGACCTCAGATCAGGCGGGACTACCCGCTGA<br>ACTTAAGCATATCAATAAGCGGAGGAAAAGAAACTAACAAGGATTC<br>CCCTAGTAACTGCGAGTGAAGCGGGAAAAGCTCAAATTTGTAATCTG<br>GCGGCCTTCGGTCGTCCGAGTTGT	*Lentinellus omphalodes*	780	780	100%	0	98%	AF506418.1
584 01 III	Qoornoq	Native dead Salix	De Novo Assemble	ACTCGGACGACCGAAGGCCGCCAGATTACAAATTTGAGCTTTTCCCG<br>CTTCACTCGCAGTTACTAGGGGAATCCTTGTTAGTTTCTTTTCCTCCGC<br>TTATTGATATGCTTAAGTTCAGCGGGTAGTCCCGCCTGATCTGAGGT<br>CAAGTTTCGATTGAAAGTGTCCCTTGCGAGACGGTTGGAAGCGGGTC<br>CCATGACATGCTAAACCCACGGCGTAGACAATTATCACACCGAGGCC<br>GCAAAGGGTCCTACTAATGCCTTCGAGAGGAGCCGAGAAGTGGGTC<br>GAAACCTTTTCCCGGCAAAAGCCTCCAAGTCCAAGCCACCGACGCCC<br>CTCGCAAAAGGGGGCGGGGTTGAGAATTTCACGACACTCAAACAGG<br>CGTACCCCTCGGAATACCAAGGGGTGCAAGGTGCGTTCAAAGATTC<br>GATGATTCACTGAATTCTGC	*Lentinellus omphalodes*	771	771	100%	0	98%	AF506418.1
592 01 I	Iffiartarfik	Native dead Salix	De Novo Assemble	TTGCAGAATTCAGTGAATCATCGAATTTTTGAACGCACCTTGCGCTCC<br>TTGGTATTCCGAGGAGCATGCCTGTTTGAGTGTCATGTTATTCTCAGC<br>CCCCTCAGATTTTGTCTCGAGGTTGGGCCTGGATTTGGAGGCTGCTG<br>GATTTTATTCAGCTCTTCTTAAATAAATTAGTGTGAATGTTCGCTGAA<br>TATCGCTCGGTGTGATAATTGTCTACACTGTCGATTTTTCGGTGAAGC<br>TAATAAAAATGTTCATGCTTCGAATTGTTCTGTTGAAGAACACTCTTA<br>TCGAATTTGACCTCAAATCAGGTAGGACTACCCGCTGAACTTAAGCA<br>TATCAATAAGCGGAGGAAAAGAAACTAACAAGGATTCCCCTAGTAA<br>CTGCGAGTGAAGCGGGAAAAGCTCAAATTTAAAATCTGATAGTCTTT<br>GGCTGTCCGAGTTG	*Hyphoderma medioburiense*	804	804	100%	0	99%	DQ677497.1
595 01 II	Iffiartarfik	Native dead Salix	Forward	CTCGGACGACCGAAGGCCGCCAGATTACAAATTTGAGCTTTTCCCGC<br>TTCACTCGCAGTTACTAGGGGAATCCTTGTTAGTTTCTTTTCCTCCGCT<br>TATTGATATGCTTAAGTTCAGCGGGTAGTCCCGCCTGATCTGAGGTC<br>AAGTTTCGATTGAAAGTGTCCCTTGCGAGACGGTTGGAAGCGGGTCC<br>CATGACATGCTAAACCCACGGCGTAGACAATTATCACACCGAGGCCG<br>CAAAGGGTCCTACTAATGCCTTCGAGAGGAGCCGAGA	*Lentinellus omphalodes*	501	501	100%	3.00E-138	99%	AF506418.1
			Reverse	CATCGAATCTTTGAACGCACCTTGCACCCCTTGGTATTCCGAGGGGT<br>ACGCCTGTTTGAGTGTCGTGAAATTCTCAACCCCGCCCCCTTTTGCGA<br>GGGGCGTCGGTGGCTTGGACTTGGAGGCTTTTGCCGGGAAAGGGTT<br>TCGACCCACTTCTC	*Lentinellus micheneri*	287	287	100%	5.00E-74	100%	AY513161.1

Sample no.	Site	Sample type	BLAST type	Sequence	Best BLAST	Max score	Total score	Query cover	E value	Ident	Accession
595 01 II	Iffiartarfik	Native dead Salix	Forward	GGCCGCCAGATTACAAATTTGAGCTTTTCCCGCTTCACTCGCAGTTACTAGGGGAATCCTTGTTAGTTTCTTTTCCTCCGCTTATTGATATGCTTAAGTTCAGCGGGTAGTCCCGCCTGATCTGAGGTCAAGTTTCGATTGAAAGTGTCCCTTGCGAGACGGTTGGAAGCGGGTCCCATGACATGCTAAACCCACGGCGTAGACAATTATCACACCGAGGCCGCAAAGGGTCCTACTAATGCCTTCGAGAGGAGCCGAGA	*Lentinellus omphalodes*	475	475	100%	2.00E-130	99%	AF506418.1
			Reverse	ATCGAATCTTTGAACGCACCTTGCACCCCTTGGTATTCCGAGGGGTACGCCTGTTTGAGTGTCGTGAAATTCTCAACCCCGCCCCCTTTTGCGAGGGGCGTCGGTGGCTTGGACTTGGAGGCTTTTGCCGGGAAAGGGTTTCGACCCACTTCTC	*Lentinellus micheneri*	285	285	100%	2.00E-73	100%	AY513161.1
595 01 III	Iffiartarfik	Native dead Salix	Forward	CTCGGACGACCGAAGGCCGCCAGATTACAAATTTGAGCTTTTCCCGCTTCACTCGCAGTTACTAGGGGAATCCTTGTTAGTTTCTTTTCCTCCGCTTATTGATATGCTTAAGTTCAGCGGGTAGTCCCGCCTGATCTGAGGTCAAGTTTCGATTGAAAGTGTCCCTTGCGAGACGGTTGGAAGCGGGTCCCATGACATGCTAAACCCACGGCGTAGACAATTATCACACCGAGGCCGCAAAGGGTCCTACTAATGCCTTCGAGAGGAGCCGAGA	*Lentinellus omphalodes*	501	501	100%	3.00E-138	99%	AF506418.1
			Reverse	GTGATCATCGAATCTTTGAACGCACCTTGCACCCCTTGGTATTCCGAGGGGTACGCCTGTTTGAGTGTCGTGAAATTCTCAACCCCGCCCCCTTTTGCGAGGGGCGTCGGTGGCTTGGACTTGGAGGCTTTTGCCGGGAAAGGGTTTCGACCCACTTCTCTCCTCTCCTC	*Lentinellus micheneri*	291	291	94%	5.00E-75	99%	AY513161.1
605 01 I	Ersaa	Native dead Salix	Forward	GCCAGATTTTAAATTTGAGCTTTTCCCGCTTCACTCGCAGTTACTAGGGGAATCCTTGTTAGTTTCTTTTCCTCCGCTTATTGATATGCTTAAGTTCAGCGGGTAGTCCTACCTGATTTGAGGTCAAAATGTCAGAGATATTGTCCCTTGCGAGACGGTTATAAGCAGGTTCCCATAAATTTGCTTCAAAGTCGACGAGGCATAGATAAT	*Mycena alexandri*	388	388	100%	2.00E-104	100%	KT900145.1
			Reverse	ATCCTTTGAACGCACCTTGCGCCCTTTGGTATTCCGAAGGGCATGCCTGTTTGAGTGTCATTAAATTATCAACCTTGCTCGTTTTTCCAATGAAGGCTTGGATGTGAGGGCTTGCTGGCTTCCTTCAGTGGATGGTCTGCTCCCTTTAAATGCATTAGTGGGATCTCTTGTGGACCGTCACTTGGCGTGATAATT	*Mycena monticola*	331	331	98%	3.00E-87	98%	EU846250.1
605 01 II	Ersaa	Native dead Salix	Forward	CACTCGGACGGCCACAAGGACCGCCAGATTTTAAATTTGAGCTTTTCCCGCTTCACTCGCAGTTACTAGGGGAATCCTTGTTAGTTTCTTTTCCTCCGCTTATTGATATGCTTAAGTTCAGCGGGTAGTCCTACCTGATTTGAGGTCAAAATGTCAAATATATTGTCCCTTGCGAGACGGTTATAAGCAGGTTCCCATAAATTTGCTTCAAAGTC	*Mycena alexandri*	385	385	99%	3.00E-103	99%	KT900145.1
			Reverse	GTGAATCATCGAATCTTTGAACGCACCTTGCGCCCTTTGGTATTCCGAAGGGCATGCCTGTTTGAGTGTCATTAAATTATCAACCTTGCTCGCTTTT	100 % ID and Query cover different species of *Mycena*						

Sample no.	Site	Sample type	BLAST type	Sequence	Best BLAST	Max score	Total score	Query cover	E value	Ident	Accession
605 01 II	Ersaa	Native dead Salix	Forward	CTCGGACGGCCACAAGGACCGCCAGATTTTAAATTTGAGCTTTTCCCGCTTCACTCGCAGTTACTAGGGGAATCCTTGTTAGTTTCTTTTCCTCCGCTTATTGATATGCTTAAGTTCAGCGGGTAGTCCTACCTGATTTGAGGTCAAAATGTCAGAGATATTGTCCCTTGCGAGACGGTTATAAGCAGGTTCCCATAAATTTGCTTCAAAGTCGACGAGGCATAGATAATT	*Mycena alexandri*	427	427	100%	5.00E-116	100%	KT900145.1
			Reverse	ATTCAGTGAATCATCGAATCTTTGAACGCACCTTGCGCCCTTTGGTATTCCGAAGGGCATGCCTGTTTGAGTGTCATTAAATTATCAACCTTGCTCGTTTTTCCAATGAAGGCTTGGATGTGAGGGCTTGCTGGCTTCCTTCAATGGATGGTCTGCTCCCTTTAAATGCATTAGTGGGATCTCTTGTGGACCGTCACTTGGCGTGATAATTA	*Mycena monticola*	363	363	100%	1.00E-96	98%	EU846250.1
605 01 III	Ersaa	Native dead Salix	Forward	ACACTCGGACGGCCACAAGGACCGCCAGATTTTAAATTTGAGCTTTTCCCGCTTCACTCGCAGTTACTAGGGGAATCCTTGTTAGTTTCTTTTCCTCCGCTTATTGATATGCTTAAGTTCAGCGGGTAGTCCTACCTGATTTGAGGTCAAATTGTCAAATATATTGTCCCTTGCGAGACGGTTATAAGCAGGTTCCCATAAATTTGCTTCACAGTCAA	*BLAST not succesful*						
			Reverse	TGCAGATTCAGTGATCATCGAATCTTTGAACGCACCTTGCGCCCTTTGGTATTCCGAAGGGCATGCCTGTTTGAGTGTCATTAAATTATCAACCTTG	*Mycena* sp.	171	171	100%	3.00E-39	98%	KY559350.1
					Mycena sp.	171	171	100%	3.00E-39	98%	KY462612.1
					Mycena alnetorum	171	171	100%	3.00E-39	98%	KU295552.1
613 01 I	Kangeq	Exp Arch	De Novo Assemble	CCAGATTACAACTCGGACGACAAAGCCGCCAGATTTTAAATTTGAGCTCATCCCGCTTCACTCGCAGTTACTAGGGGAATCCTTGTTAGTTTCTTTTCCTCCGCTTATTGATATGCTTAAGTTCAGCGGGTAGTCCTACCTGATTTGAGATCGGATTTCAAAAGGTTGTCCGAAGACGGTTAGAAGCTCGACCGACTCGAGGTCCCATGGGTGTAGATAATTATCACACCGTGAACCGCCGTGCCGTGGCGCCCAGCTAATGCATTCAAGGGGAGCCGATCGCTCGACGCGACCGGCAATGAACCCCCAACTCCAAGCCACCCACAAACAAATGCGGGGGTTGATGATTTCATGATACTCAAACAGGCATGCTCCTCGGAATACCAAGGAGCGCAAGGTGCGTTCAAAGATTCGATGATTCACTGAATTCTGCAA	*Uncultured fungus clone*	628	628	100%	3.00E-176	92%	EU292344.1
					Uncultured fungus isolate from decayed roots of conifer	551	551	68%	6.00E-153	100%	DQ093750.1
					Uncultured fungus clone	529	529	70%	3.00E-146	98%	KU189026.1

Sample no.	Site	Sample type	BLAST type	Sequence	Best BLAST	Max score	Total score	Query cover	E value	Ident	Accession
625 01 II	Kangeq	Exp Hist	Forward	GATTACACTCGGATGATCAAAGACCACCAGATTTTAAATTTGAGCTTT<br>TCCCGCTTCACTCGCAGTTACTAGGGGAATCCTTGTTAGTTTCTTTTCC<br>TCCGCTTATTGATATGCTTAAGTTCAGCGGGTAGTCCTACCTGATTTG<br>AGGTCAAATTGTCATTATTTGTCCGACTTAACAGACGGTTAGAAGCA<br>GTACAATCCATTTAAAGTGAAGGCCCACGGCGTAGATAATTATCACA<br>CCAATAGACGACTCCACACGGGTACCGGCTAATACATTTAAGGGGA<br>GCAGACCTCTTGACGAAGCCAGCAAAATACCCCCAAGTCCAAGCCAT<br>TACCAATTTGTGAAAACTGGTAAGGTTGAGAATTTAATGACACTCAA<br>ACAGGCATGCTCCTCGGAATACCAAGGAGCGCAAGGTGCGTTCAAA<br>GATTCGATGATTCACTGAATTCTGCAATTCACATTACTTATCGCATTTC<br>GCTGC	*Galerina marginata*	863	863	100%	0	99%	KX236118.1
			Reverse	-							
625 01 III	Kangeq	Exp Hist	De Novo Assemble	GTGAATCATCGAATCTTTGAACGCACCTTGCGCTCCTTGGTATTCCGA<br>GGAGCATGCCTGTTTGAGTGTCATTAAATTCTCAACCTTACCAGTTTT<br>CACAAATTGGTAATGGCTTGGACTTGGGGGTATTTTGCTGGCTTCGT<br>CAAGAGGTCTGCTCCCCTTAAATGTATTAGCCGGTACCCGTGTGGAG<br>TCGTCTATTGGTGTGATAATTATCTACGCCGTGGGCCTTCACTTTAAA<br>TGGATTGTACTGCTTCTAACCGTCTGTTAAGTCGGACAAATAATGAC<br>AATTTGACCTCAAATCAGGTAGGACTACCCGCTGAACTTAAGCATAT<br>CAATAAGCGGAGGAAAAGAAACTAACAAGGATTCCCCTAGTAACTG<br>CGAGTGAAGCGGGAAAAGCTCAAATTTAAAATCTGGTGGTCTTTGAT<br>CATCCGA	*Galerina marginata*	782	782	100%	0	99%	KX236118.1

Supplementary Methods S10

Visual decay tests, light microscopy

Samples were observed by light microscopic examination of cross sections to determine an average degree of degradation as either intact, partly decayed, or in a terminal stage of decay.

Intact xylem: Cell walls and compound middle lamella of tracheids and parenchyma cells were morphological intact. Few indications of incipient decay were allowed in this category; e.g. incipient decay of parenchyma cells in rays.

Partly decayed xylem: The xylem contains a varying degree of decayed and intact wood cells. Either as larger areas of intact xylem or as cell walls that are only partly decayed throughout the sample.

Terminal decay stage: All wood cells are decayed to the terminal stage of the specific dominant decay type; no food source is left for the dominant decay type present in the wood. Or the wood xylem is decayed to a stage where it is not possible to cut hand section as the xylem falls apart; often fine roots are penetration the xylem.

Visual decay tests, scanning electron microscopy

Nine buried archaeological samples were selected for scanning electron microscopy analysis. Samples were prepared for scanning electron microscopy (SEM) by infiltrating with a 25 % TBS Tissue Freezing Medium (Triangle Biomedical Sciences, Durham, NC, U.S.A.) under vacuum followed by mounting on brass stubs, freezing at -20 °C and sectioning in a cryostat freezing microtome. Samples were then cut transversely to prepare a clean surface for examination. Cut samples were thawed and rinsed several times in water, air dried before mounting on aluminium stubs with carbon tape and coated with gold using a sputter coater. Samples were viewed using a Hitachi S3500N scanning electron microscope to determine characteristics of decay and signatures of fungal colonization in the cell wall structure.

Culturing media

A wood culture media were prepared by mixing 12.0 g agar (Sigma), 4.0 g milled (Retsch SM 2000, mesh 0.5 mm) *Pinus sylvestris* drift wood collected in the Disco Bay, Greenland in 2015, 100 mg KCl (Fluka), 200 mg KH_2PO_4 (J.T. Baker), 100 mg $MgSO_4$ • 7 H2O (Sigma-Aldrich), 200 mg $Ca(NO_3)_2$ • 7 H2O, 100 mg 2xYT yeast extract tryptone (Difco), and 50 mg Novobiocin sodium salt (Sigma) to suppress bacterial growth in one liter of deionized water. The same media was also prepared with addition of 2.0 g D(+)-glucose (Merck) and with addition of 20.0 mg Benomyl (Sigma) to suppress Ascomycota growth. Malt extract agar was prepared by mixing 48.0 g malt extract agar (Merck) with 50 mg Novobiocin sodium salt (Sigma) in one liter of deionized water. The same media was also prepared with addition of 20.0 mg Benomyl (Sigma). A Basidiomycota specific agar was prepared by mixing 24.0 g malt extract agar (Merck), 7.5 g agar, 2.0 g 2xYT yeast extract tryptone (Difco), 50 mg

Novobiocin sodium salt (Sigma), 60 mg Benomyl (Sigma), and 2.0 mL 85 % lactic acid in one liter of deionized water. All culture media were autoclaved at 125 °C for 20 minutes and poured into plates; with the exception, that lactic acid was added to the Basidiomycota specific agar with sterile pipette after autoclaving and cooling to approximate 50 °C.

qPCR, DNA sample preparation

Experimental samples: Undiluted, 10×, 100×, 1,000× and 10,000× diluted experimental sample concentrations and primer concentration were tested to investigate the presence of compounds inhibitory (impurities) to PCR amplification and to ensure that the amount of template fell in the linear range of the standard curves. A primer concentration of 200 nM and 1000× dilution of the DNA extraction showed no indications of inhibition in the majority of the samples. However, six samples were diluted 10.000x to avoid inhibition. Finally each subsample was prepared by mixing 2.00 µL diluted isolated gDNA, 200 nM forward DNA_{bas} primer, 200 nM reverse DNA_{bas} primer, 5.00 µL Fast SYBR Green master mix (Applied Biosystems, Waltham, MA, USA), and 2.60 µL nuclease free water and amplified. The subsamples were then prepared once again by mixing 2.00 µL diluted isolated gDNA, 300 nM forward pGEMF primer, 300 nM reverse pGEMR primer, 300 nM FAM probe pGEMT, 5.00 µL TaqMan Fast Universal PCR Master mix (2X) (Applied Biosystems, MA, USA), and 2.01 µL nuclease free water and amplified.

Basidiomycete standard curve: A stock solution for a gDNA standard curve were prepared by mixing 1064 ng isolated gDNA from *Trametes versicolor* with 1064 ng isolated gDNA from *Postia placenta* in nuclease free water with a final concentration of 45,1 ng gDNA/µl. This stock solution was diluted to give eight steps each diluted 10 times in nuclease free water from 3 ng to $3\cdot10^{-7}$ ng gDNA.

Reference gene standard curve: The 0.01 ng pGEM/µl. stock solution (pGEM-3Z Vector, Promega, Madison, WI, USA) was diluted 10 times from 0.02 ng to $2.0\cdot10^{-9}$ ng.

Identification of Basidiomycota in gDNA extracted directly from wood samples, mastermix

A mastermix (in total 48 µL for each subsample) was prepared containing 5.00 µL 10x PCR buffer, 2.00 µL 25 mM $MgCl_2$, 1.00 µL dNTP mix, 5.00 µL 0.4% BSA, 5.00 µL 0.1 mM TMACl and 0.40 µL HotStarTaq Plus DNA polymerase; all from Qiagen GmbH, Hilden, Germany. In addition 2.00 µM forward primer (5.8sr) and 2.00 µM reverse primer (ITS4-X) (Applied Biosystems, Waltham, MA, USA) and 27.6 µL nuclease free water.

Identification of Basidiomycota in gDNA extracted directly from wood samples, PCR amplification

Polymerase chain reactions were performed using the Applied Biosystems GeneAmp PCR System 9700 (Applied Biosystems, Waltham, MA, USA) running with an initial activation step at 95°C for 5 minutes followed by 35 PCR cycles running with denaturation at 95 °C for

30 seconds, annealing at 60 °C for 30 seconds, and 1 minute of extension at 72 °C. The 35 PCR cycles were followed by 10 minutes of final extension at 72 °C and cooling at 4 °C until removed from the thermocycler. The size and quality of the PCR products were verified by electrophoresis on a 1% agarose gel with 14 µL ethildiumbromid 1 % stain (VWR International) and trans-illumination with a Syngene (Syngene, Cambridge, UK): The PCR products were run at agarose gels to determine purity and size of the products. 140 mL 1x Tris-acetate-EDTA (TAE) buffer was mixed with 1.4 g SeaKem LE Agarose (Lonza, Basel, Switzerland) and cooked in microwave oven until dissolved. The gel was cooled to 60 °C while shaken gently. 14 µL ethildiumbromid 1 % (VWR International, Radnor, PA, USA) was added and gently mixed before poured into a 20 x 2 well mould and air bobbles removed with a pipette tip. The gel was transferred to a electrophorese basin with TAE buffer. 7.0 µL PCR product from each sample was mixed with 1.5 µL 10x gel loading buffer dye (Invitrogen, Waltham, MA, USA) and loaded into separate wells. Two wells were loaded with 1 Kb Plus DNA Ladder (Invitrogen by Life Technologies, Waltham, MA, USA) diluted to 0.10 µg/µL. The electrophoresis was initial run at 80 V until the PCR products had moved into the gel hereafter at 100 V for 30 minutes. The agarose gel was trans-illuminated with a Syngene (Syngene, Cambridge, UK).
